# A pH-Dependent Curcumin–Iminosugar Hybrid Promotes GCase Enhancement and Reactive Oxygen Species Reduction

**DOI:** 10.1021/acsomega.6c08401

**Published:** 2026-10-01

**Authors:** Alessio Morano, Camilla Matassini, Francesca Cardona, Damiano Tanini, Silvia Falliano, Amelia Morrone, Rebecca Sodano, Paolo Paoli, Andrea Goti, Francesca Clemente

**Affiliations:** † Department of Chemistry “Ugo Schiff” (DICUS), 9300 University of Florence, Via della Lastruccia 3−13, Sesto F.no (FI) 50019, Italy; ‡ Laboratory of Molecular Genetics of Neurometabolic Diseases, Department of Neuroscience and Medical Genetics, Meyer Children’s Hospital IRCCS, Viale Pieraccini 24, Firenze 50139, Italy; § Department of Neurosciences, Psychology, Drug Research and Child Health (NEUROFARBA), University of Florence, Viale Pieraccini 24, Firenze 50139, Italy; ∥ Department of Experimental and Clinical Biomedical Sciences (DSBSC), University of Florence, Viale Morgagni 50, Firenze 50134 Italy

## Abstract

Gaucher disease and Parkinson’s disease are characterized by β-glucocerebrosidase (GCase) dysfunction and increased oxidative stress. Here, we report two multifunctional hybrids that combine an iminosugar-based pharmacological chaperone scaffold with curcumin- or ferulic acid-derived antioxidant motifs. The curcumin-iminosugar hybrid **2** exhibits pH-dependent GCase modulation, enhanced GCase activity under lysosomal conditions, enhanced enzyme activity in L444P Gaucher and wild-type fibroblasts, and reduced intracellular reactive oxygen species (ROS) levels. By integrating GCase enhancement and redox modulation within a single molecular framework, compound **2** represents a promising lead for Gaucher disease and GCase-related synucleinopathies.

## Graphical abstract

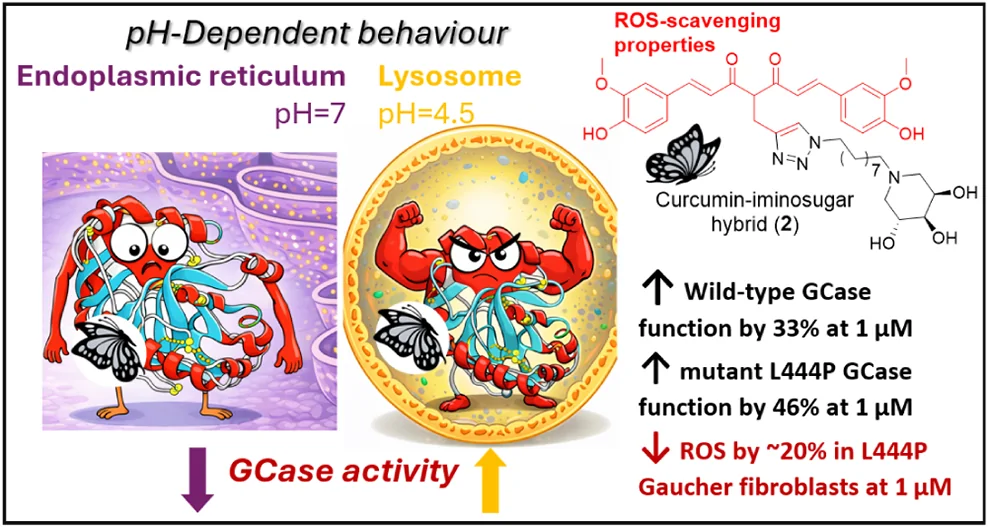

## Introduction

Curcumin (**1**), a natural polyphenol isolated from the rhizome of *Curcuma longa*, has attracted significant attention owing to its antioxidant and redox-modulating properties. Structurally, it consists of two guaiacol (*o*-methoxyphenol) moieties linked through a vinylogous β-diketone framework, a feature that enables the efficient neutralization of reactive oxygen and nitrogen species (ROS/RNS) and the modulation of endogenous antioxidant defenses.^1^ Recent studies have further highlighted its ability to activate cytoprotective signaling pathways in addition to direct radical scavenging activity,^2–5^ supporting its therapeutic potential in disorders driven by oxidative stress, including neurodegenerative diseases, metabolic syndromes and cancer.^6–11^

In our field of interest, particularly in the context of Gaucher disease, increasing attention has been directed toward multitarget strategies, which are considered especially interesting due to their potential to concurrently address distinct pathogenic pathways.^12,13^ Gaucher disease (GD) results from mutations in the *GBA1* gene, which encodes β-glucocerebrosidase (GCase). These mutations lead to deficient enzyme activity, causing glucosylceramide accumulation, lysosomal dysfunction and elevated oxidative stress.^14^ Increased levels of inflammation and oxidative stress markers have been consistently reported in both experimental animal models and patients with GD.^15–21^ Among small-molecule approaches aimed at restoring GCase activity, the development of pharmacological chaperones (PCs) has emerged as a particularly promising strategy. PCs bind selectively to misfolded defective GCase in the endoplasmic reticulum (ER), correcting its conformation and promoting correct trafficking to the lysosome.^22^ A typical class of PCs is represented by iminosugars, carbohydrate mimetics in which the endocyclic oxygen is replaced by a nitrogen atom.^23,24^ This approach becomes even more relevant when considering the strong mechanistic link between GD and Parkinson disease (PD), since mutations in the *GBA1* gene are among the major genetic risk factors for PD.^25–27^ Moreover, it is now well established that a reduction in wild-type (WT) GCase activity is also observed in sporadic PD cases even in the absence of *GBA1* mutations.^28,29^ Decreased GCase functioning promotes α-synuclein accumulation, lysosomal impairment and increased oxidative stress, creating a pathogenic feedback loop.^30,31^

Therefore, therapeutic strategies exclusively focused on targeting GCase activity may not be sufficient. While GCase activation may indirectly mitigate oxidative stress and α-synuclein accumulation, our goal was to develop multifunctional compounds that combine GCase enhancement with intrinsic antioxidant properties^32^ or direct modulation of α-synuclein,^33^ thereby addressing multiple interconnected pathological pathways simultaneously. In the present work, we aimed to develop multifunctional PCs capable of combining GCase stabilization with antioxidant activity. To this end, we designed hybrid **2** by conjugating a validated *N*-alkyl-3,4,5-trihydroxypiperidine PC scaffold,^32,34,35^ a known GCase stabilizer, with curcumin, thereby coupling chaperone activity and redox modulation within a single molecular framework (Figure 1). In parallel, we synthesized analogue **3** by conjugation with a ferulic acid moiety (Figure 1). As a naturally occurring phenolic antioxidant, ferulic acid can be viewed as a structurally simplified “half-curcumin” surrogate that retains the key radical-scavenging features of curcumin while reducing molecular complexity.^36,37^

**Figure 1:**
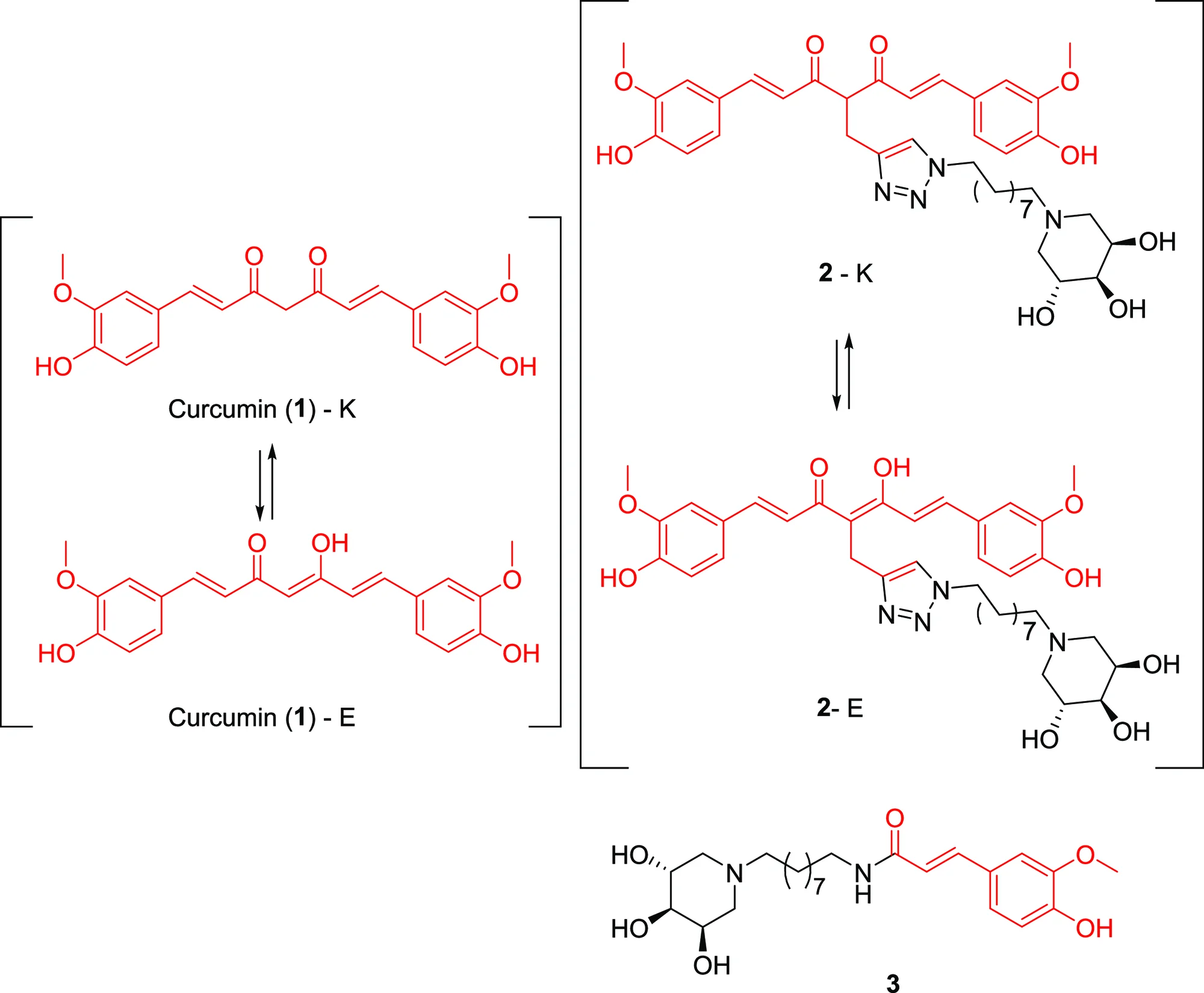
Structures of Curcumin (**1**) and new iminosugar curcumin **2** and iminosugar-ferulic acid **3** hybrids.

The (*R*,*R*)-3,4,5-trihydroxypiperidine was converted to azide **4** as described in the literature,^34^ enabling conjugation with a terminal alkyne-armed curcumin derivative **6** prepared as reported.^38^ The coupling was performed via copper-catalyzed azide–alkyne cycloaddition (CuAAC) using CuSO_4_/sodium ascorbate in THF/H_2_O (2:1) until complete conversion of the curcumin derivative (which occurred after 23 h at room temperature). After treatment with QuadraSil MP and purification by column chromatography, the hybrid **2** was obtained in 41% yield as a mixture of two tautomers (Scheme 1). Alternatively, amine **5** ^32^ was coupled with ferulic acid (**7**) under standard EDC/HOBt conditions in dry DMF. Hybrid **3** was isolated in 42% yield after 16 h at room temperature and purification by silica gel chromatography (Scheme 1).

**Scheme 1:**
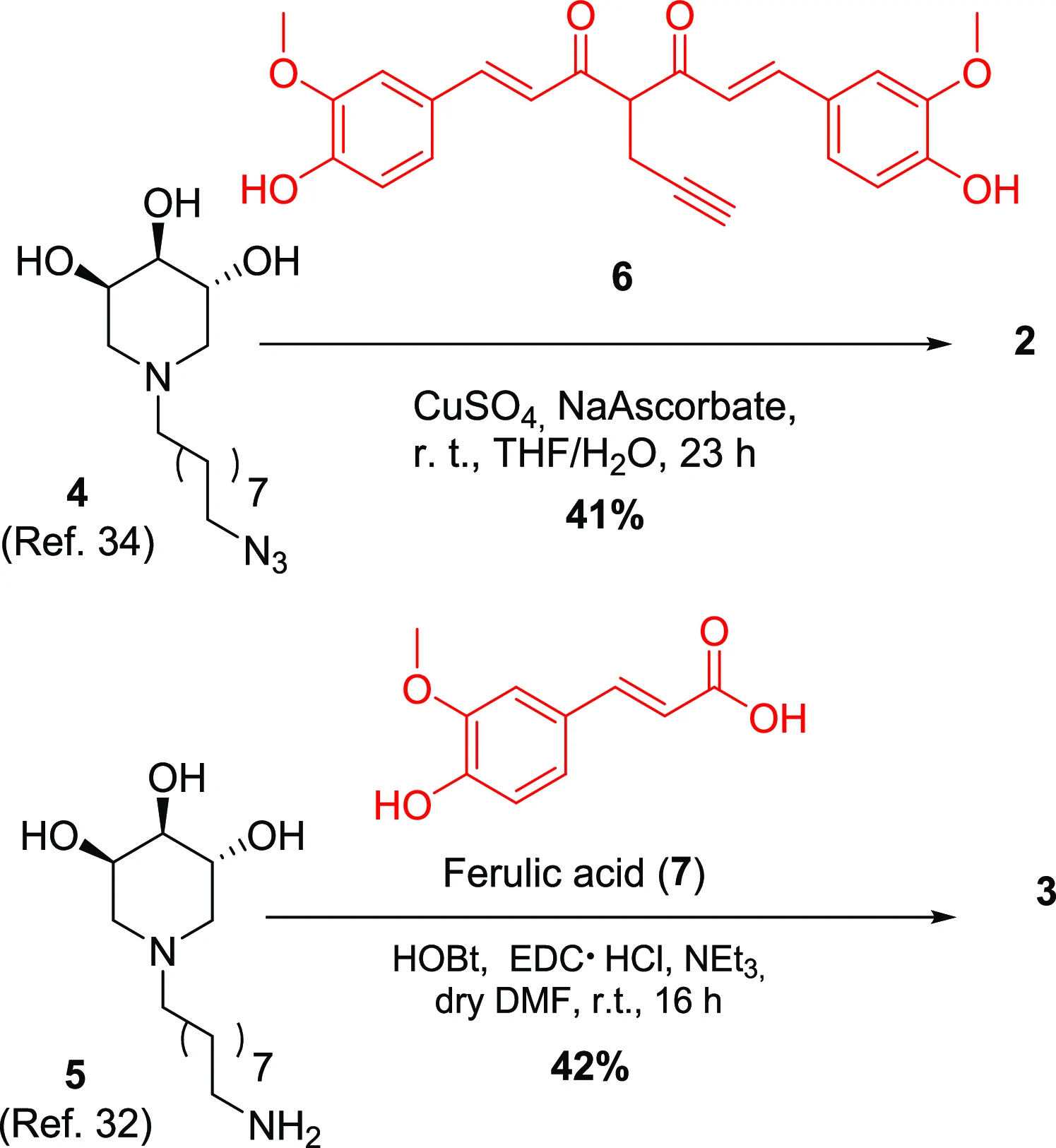
Synthesis of Iminosugar-Curcumin **2** and Iminosugar-Ferulic Acid **3** Hybrids

Preliminary biological evaluation of the hybrids was performed by measuring their inhibitory activity against recombinant human GCase (rh-GCase) at pH 4.5 and 7.0, mimicking lysosomal and ER conditions, respectively. For comparison, iminosugar **8**, previously identified as our reference GCase piperidine-type PC^39^ and curcumin (**1**; Supelco, 08511–10MG), were evaluated under identical experimental conditions (Table 1). At pH 7.0, all compounds behaved as inhibitors of rh-GCase. Compounds **2** (IC_50_ = 12.0 ± 2.4 μM), **3** (IC_50_ = 20 ± 1 μM) and **8** (IC_50_ = 21.0 ± 3.3 μM) exhibited moderate inhibitory potency in the low micromolar range, whereas curcumin (**1**) was significantly less active (IC_50_ ≈ 500 μM) (Table 1). These data indicate that the hybrids **2** and **3** retain the typical inhibitory profile of iminosugar-based scaffolds under neutral conditions, where binding to the enzyme in the ER is required to stabilize its folded conformation, a prerequisite to a potential PC mechanism. In contrast, a different behavior was observed at pH 4.5. Hybrid **2** induced a marked enhancement of enzyme activity, reaching up to 2.8 fold at 30 μM, suggesting an enhancer effect under lysosomal conditions. Curcumin (**1**) also promoted enzyme activation (∼2 fold), with a more modest effect, suggesting that the iminosugar-containing compound may provide a more effective enhancement of enzyme activity. In contrast, **3** and **8** displayed a weak enzyme inhibition (IC_50_ ∼500 μM and ∼2000 μM, respectively), reflecting a substantial loss of inhibitory potency compared to pH 7.0 (Table 1). This pronounced pH-dependent behavior is a key requirement for effective PCs. Indeed, the use of inhibitory chaperones, that continue to bind and inhibit the lysosomal enzymes once they have reached the lysosomes, represents the main challenge for their clinical development. Remarkably, the compounds reported herein bind the enzyme strongly in the ER at neutral pH but lose affinity under acidic lysosomal conditions or even enhance catalytic activity. The observed loss of inhibition and, in the case of **1** and **2**, enzyme activation at pH 4.5 therefore represents a highly favorable functional profile. To the best of our knowledge, although GCase activators have been described previously^40–43^ and have recently been discussed in the context of the emerging landscape of small-molecule GCase modulators,^44^ none have been reported to display the pronounced pH-dependent switch from inhibition at neutral pH to activation under lysosomal acidic conditions observed for compounds **1** and **2**. Kinetic studies were performed to investigate their mechanism of action, revealing distinct behaviors for compounds **1** and **2**, likely arising from their different chemical architectures (see Supporting Information). Curcumin (**1**) increased GCase activity without affecting Km, consistent with a nonessential activator mechanism likely mediated by binding to a secondary site and inducing conformational changes that enhance catalysis. In contrast, hybrid **2**, which combines a curcumin moiety with an *N*-alkylated 3,4,5-trihydroxypiperidine unit, increased both Km and Vmax, suggesting simultaneous engagement of an activation site and the active site. Notably, **2** showed stronger activation than **1** and greater inhibitory potency than 3,4,5-trihydroxypiperidine alone, supporting a cooperative interplay between the two pharmacophores. Although a contribution from pH-dependent differences in stability or solubility cannot be excluded, the distinct kinetic behavior of **2** compared with curcumin alone supports a direct contribution of the iminosugar moiety to GCase interaction. These findings highlight the potential of dual-binding molecules as GCase enhancers.

**Table 1:**
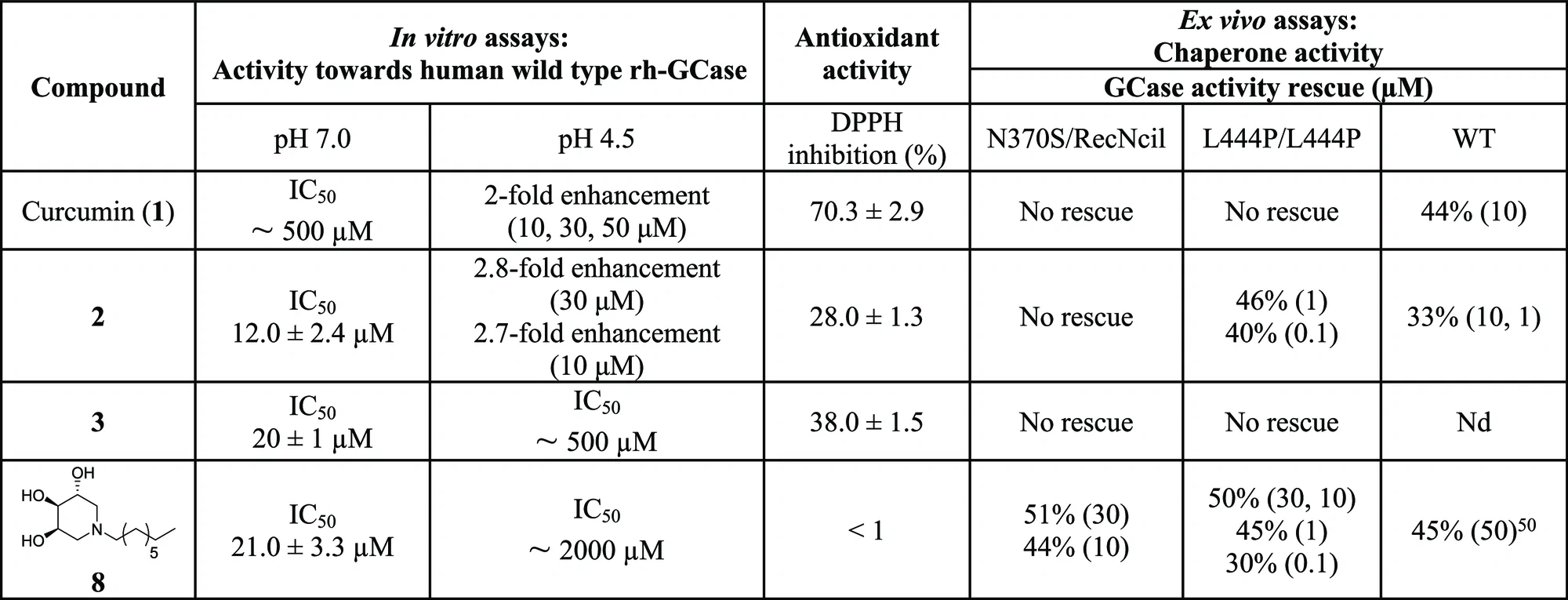
Summary of the Biological Evaluation of the Investigated Compounds: *In Vitro* GCase Interaction at pH 7.0 and 4.5, Expressed as IC_50_ Values for Inhibitory Activity or as Fold Enhancement Relative to the Control (in the Absence of Compounds), *In Vitro* Antioxidant Activity and *Ex Vivo* PC Activity in WT and GD Patient-Derived Fibroblasts (Nd: Not Determined)

The chain-breaking antioxidant activity of the synthesized hybrid molecules was assessed using the 2,2-diphenyl-1-picrylhydrazyl (DPPH) radical assay in ethanol.^45^ The time course of DPPH decolorization by an equimolar amount of the tested compounds allows determination of the percentage of DPPH inhibition (Table 1, see Supporting Information). The curcumin hybrid **2** and the feruloyl amide derivative **3** exhibited comparable radical scavenging properties in this assay, while curcumin (**1**) showed higher radical scavenging activity. The percentage of DPPH inhibition determined 10 min after the addition of the test compounds, widely considered a benchmark time point for assessing radical scavenging activity, was 70.3% for curcumin. On the other hand, compounds **2** and **3** exhibited DPPH inhibition values of 28% and 38%, respectively. For comparison purposes, the activity of **8** (not containing curcumin- or ferulic acid-related moieties) was also evaluated and, as expected, no radical scavenging activity was observed. All derivatives investigated herein exhibited lower antioxidant activity than di­(*tert*-butyl)­hydroxytoluene (BHT), employed as the positive control, which showed more than 95% inhibition of the DPPH radical.

To further evaluate the therapeutic potential of the compounds, experiments were conducted in patient-derived fibroblasts carrying either the N370S/RecNcil or the L444P/L444P *GBA1* mutations, two of the most common variants associated with GD^46^ and *GBA1*-related PD.^27^ Cells were treated with increasing concentrations of each compound for 4 days and GCase activity was measured in intact cells.^32,47^ As reported, the reference PCs Ambroxol (ABX) and Isofagomine (IFG) significantly increased GCase activity in N370S mutant fibroblasts but failed to rescue enzyme activity in L444P cells,^48,49^ confirming the well-known mutation-dependent effect of PCs. Compounds **1**-**3** and **8** were evaluated under the same experimental conditions (see Supporting Information). Curcumin (**1**) and the feruloyl amide **3** did not show significant activity in either genotype (Table 1). The *N*-octylpiperidine **8** demonstrated a consistent and dose-dependent increase in GCase activity in both genotypes, suggesting a broad activity profile that is not limited to specific mutations and confirming its excellent PC performance, even when evaluated with this intact-cell method (Table 1). Interestingly, the curcumin-iminosugar hybrid **2**, while being inactive in N370S mutated cells, produced a considerable increase in GCase activity in L444P fibroblasts at low concentrations (46% at 1 μM and 40% at 0.1 μM) (Table 1). It is important to note that the L444P genotype represents one of the most severe neuropathic forms and is typically considered highly resistant to PC treatment.^23,24^ Therefore, the ability of **2** to enhance GCase activity in this particularly refractory context is especially noteworthy. In contrast, curcumin (**1**) alone and the “half-curcumin” hybrid **3** did not show any significant PC effect under the tested conditions. This lack of activity suggests that the intact hybrid curcumin structure **2** is essential for effective GCase stabilization and restoration of enzyme function. To assess their potential relevance to sporadic PD, we evaluated the effects of **1** and **2** in wild-type human fibroblasts. As shown in Table 1, compounds **2** (at 1 and 10 μM), and **1** (at 10 μM), increased GCase activity by 33% and 44%, respectively (see Supporting Information), supporting their potential for further investigation in sporadic PD. Notably, a comparable enhancement of GCase activity (∼45%) has been reported for iminosugar-based chaperone **8** only at significantly higher concentrations (50 μM),^50^ highlighting the potency of **1** and **2**. Having demonstrated potent PC activity, compound **2** was subsequently assessed for its ability to mitigate intracellular ROS accumulation. Remarkably, at the same concentration that showed significant PC activity, **2** achieved an approximate 20% decrease in ROS levels in L444P fibroblasts (see Supporting Information), demonstrating a clear antioxidant effect. This complementary action, simultaneously enhancing enzymatic performance while alleviating oxidative stress, further emphasizes the therapeutic promise of compound **2** as a multifunctional candidate for the treatment of GD and *GBA1*-related PD.

In summary, we developed two novel iminosugar-based hybrids incorporating antioxidant motifs and evaluated their potential as multifunctional modulators of GCase. Among them, the curcumin-derived hybrid **2** displayed a unique and highly favorable profile, combining pH-dependent GCase modulation, enzyme enhancement under lysosomal conditions, PC activity in L444P GD and in WT fibroblasts, and a significant reduction of intracellular ROS levels. These findings identify compound **2** as a promising dual-target lead for GD and GBA1-associated PD, supporting the development of multifunctional therapeutic strategies that simultaneously address enzyme dysfunction and oxidative stress.

## Methods

### General Procedures

Commercial reagents were used as received. All reactions were magnetically stirred and monitored by TLC on 0.25 mm silica gel plates (Merck F254) and column chromatography were carried out on Silica Gel 60 (32–63 μm). Yields refer to spectroscopically and analytically pure compounds unless otherwise stated. NMR spectra were recorded on a Varian Mercury-400, Varian INOVA-400 or Varian Gemini-200. The notations of multiplicity (s, d, t, q) in the ^13^C NMR spectra do not derive from a decoupled experiment but are deduced on the basis of signal attribution through 2D spectra. For practical reasons the assignment of H and C atoms in NMR characterizations reflects the numbering of chemical structures. Infrared spectra were recorded with a PerkinElmer Spectrum BX FT-IR System spectrophotometer. ESI full MS were recorded on a Thermo LTQ instrument by direct inlet; relative percentages are shown in brackets. Elemental analyses were performed with a PerkinElmer 2400 analyzer. Optical rotation measurements were performed with a JASCO DIP-370 polarimeter.

Compound **8** employed in the biological assays had previously been synthesized following the established laboratory procedure, while commercial curcumin (**1**) was obtained as an analytical standard (Curcumin, Supelco, 08511-10MG).

### Synthesis of Compound **2**

To a solution of curcumin derivative **6** ^38^ (61 mg, 0.150 mmol) and piperidine **4** ^34^ (59 mg, 0.195 mmol) in THF (3.6 mL) and H_2_O (1.8 mL), CuSO_4_·5H_2_O (7 mg, 0.0439 mmol) and Na Ascorbate (15 mg, 0.0757 mmol) were added. The reaction mixture was stirred in the dark for 23 h at room temperature, until a TLC control (PE/EtOAc 1:1) attested the disappearance of the starting material. The mixture was filtered through Celite and the solvent was removed under reduced pressure. The crude product was first treated with QuadraSil MP resin and then purified on silica gel by flash column chromatography in the dark (gradient of eluent from CH_2_Cl_2_/MeOH/NH_4_OH (6%) 10:1:0.1 to 5:1:0.1) to afford the product **2** as a mixture of tautomers (44 mg, 0.0622 mmol, 41%).

**2**: red solid mp 103.9–105.1 °C. Rf = 0.2 (CH_2_Cl_2_/MeOH/NH_4_OH (6%) 5:1:0.1). Mixture of tautomers (K + E): ^1^H NMR (400 MHz, CD_3_OD) δ ppm: 7.74–7.50 (m, 2H, Ar and conjugated double bonds, K + E), 7.47–7.30 (m, 1H, Ar and conjugated double bonds, K + E), 7.23–6.73 (m, 8H, Ar and conjugated double bonds, K + E), 5.50–5.42 (m, 1H, H-4′, K), 4.36–4.21 (m, 2H, CH_2_-N­(triazole)), 3.97–3.77 (m, 8H, OCH_3_, H-3, H-5; K + E), 3.47–3.39 (m, 1H, H-4, K + E), 3.12–3.01 (m, 2H, CH_2_-(triazole), E), 2.87–2.73 (m, 4H, Ha-6, Ha-2 K + E, CH_2_-(triazole) K), 2.48–2.27 (m, 3H, Hb-2, CH_2_-piperidine, K + E), 2.21–2.11 (m, 1H, Hb-6, K + E), 1.85–1.69 (m, 2H, CH_2_CH_2_–triazole, K + E), 1.53–1.37 (m, 2H, CH_2_CH_2_–piperidine, K + E), 1.32–1.11 (m, 10H, CH_2_, K + E). ^13^C NMR (100 MHz, CD_3_OD) δ ppm: 196.4, 194.4, 168.1, 151.8, 150.3, 149.5, 149.4, 149.4, 149.2, 148.8, 148.3, 147.0, 143.6, 140.5, 131.5, 128.9, 127.3, 125.3, 124.4, 124.3, 123.8, 123.5, 123.1, 120.8, 116.7, 116.7, 116.6, 116.6, 116.2, 113.9, 112.0, 111.7, 111.5, 81.0, 80.9, 75.0, 69.4, 68.8, 59.2, 57.9, 57.3, 56.5, 56.4, 51.3, 43.3, 40.4, 31.2, 31.8, 30.8, 30.7, 30.4, 30.4, 30.3, 29.9, 29.9, 28.4, 27.4, 27.4, 27.3, 25.6, 20.6. MS (ESI): *m*/*z* (%) = 707.33 (100) [M + H]^+^. C_38_H_50_N_4_O_9_ (706.36): calcd C, 64.57; H, 7.13; N, 7.93; found C, 64.56%; H, 7.19%; N, 7.66%.

### Synthesis of Compound **3**

To a solution of piperidine **5** ^32^ (0.241 mmol, 66 mg) in dry DMF (3 mL), ferulic acid (**7**, 0.219 mmol, 43 mg), *N*-hydroxybenzotriazole (HOBt, 0.241 mmol, 33 mg), 1-ethyl-3-(3-(dimethylamino)­propyl)­carbodiimide hydrochloride (EDC·HCl, 0.241 mmol, 46 mg) and triethylamine (0.241 mmol, 34 μL) were added under nitrogen atmosphere. The reaction mixture was stirred in the dark at room temperature under nitrogen atmosphere for 16 h, until a TLC control (CH_2_Cl_2_/MeOH/NH_4_OH (6%) 5:1:0.1) attested the disappearance of the carboxylic acid. The crude product was purified on silica gel by flash column chromatography (washed with PE and then gradient of eluent from CH_2_Cl_2_/MeOH/NH_4_OH (6%) 10:1:0.1 to 5:1:0.1) to afford the product **3** (41 mg, 0.0910 mmol, 42%).

**3**: yellow solid mp 58.6–60.4 °C. Rf = 0.1 (CH_2_Cl_2_/MeOH/NH_4_OH (6%) 10:1:0.1). [α]_D_^24^ = −13.2 (c = 0.5, MeOH). ^1^H NMR (400 MHz, CD_3_OD) δ ppm: 7.45 (d, J = 15.7 Hz, 1H, H-3′), 7.14 (d, J = 1.8 Hz, 1H, H-5′), 7.04 (dd, J = 8.2, 1.8 Hz, 1H, H-9′), 6.81 (d, J = 8.2 Hz, 1H, H-8′), 6.45 (d, J = 15.7 Hz, 1H, H-2′), 3.99–3.94 (m, 1H, H-3), 3.90 (s, 3H, OCH_3_), 3.89−3.83 (m, 1H, H-5), 3.54–3.44 (m, 1H, H-4), 3.34–3.27 (m, 2H, CH_2_-NH), 2.97–2.83 (m, 2H, Ha-2, Ha-6), 2.63–2.44 (m, 3H, Hb-2, CH_2_-piperidine), 2.44–2.26 (m, 1H, Hb-6), 1.68–1.48 (m, 4H, CH_2_CH_2_–NH, CH_2_CH_2_–piperidine), 1.48–1.24 (m, 10H, CH_2_). ^13^C NMR (100 MHz, CD_3_OD) δ ppm: 169.1 (s, C=O), 149.8 (s, Cq), 149.3 (s, Cq), 141.9 (d, H-3′), 128.3 (s, Cq), 123.2 (d, H-9′), 118.8 (d, H-2′), 116.5 (d, H-8′), 111.6 (d, H-5′), 74.1 (d, C-4), 69.1 (d, C-5), 68.2 (d, C-3), 59.0 (t, CH_2_-piperidine), 57.1 (t, C-6), 56.8 (t, C-2), 56.4 (q, OCH_3_), 40.5 (t, CH_2_–NH), 30.5 (t, CH_2_), 30.5 (t, CH_2_), 30.4 (t, CH_2_), 30.3 (t, CH_2_), 28.3 (t, CH_2_), 28.0 (t, CH_2_), 26.9 (t, CH_2_). MS (ESI): *m*/*z* (%) = 451.33 [M + H]^+^ (100), 473.25 (68) [M + Na]^+^, 922.92 (97) [2M+Na]^+^. C_24_H_38_N_2_O_6_ (450.27): calcd C, 63.98; H, 8.50; N, 6.22; found C, 63.91%; H, 8.83%; N, 6.24%.

### Activity toward Human GCase

All experiments on biological materials were performed in accordance with the ethical standards of the institutional research committee and with the 1964 Helsinki Declaration and its later amendments.

Compounds **1**, **2** and **3** were dissolved in DMSO and used immediately. For each replicate, freshly dissolved compound from the same synthesis batch was used. Compound **8** was dissolved in water.

All compounds were screened against recombinant human GCase (rh-GCase; Cerezyme) at pH 4.5 and 7.0, which mimic the lysosomal and endoplasmic reticulum environments, respectively.

Enzyme activity was measured in a flat-bottomed 96-well plate. Compound solution (3 μL), rh-GCase (7 μL), and substrate 4-methylumbelliferyl-β-d-glucoside (3.33 mM, 20 μL, Sigma-Aldrich) in citrate/phosphate buffer at pH 4.5 and 7.0 containing sodium taurocholate (0.3%) and Triton X-100 (0.15%) at 37 °C were incubated for 25 min. The reaction was stopped by the addition of sodium carbonate (200 μL; 0.5 M, pH 10.7) containing Triton X-100 (0.0025%), and the fluorescence of 4-methylumbelliferone released by β-glucosidase activity was measured in SpectraMax M2 microplate reader (λ_ex_ = 365 nm, λ_em_ = 435 nm; Molecular Devices). GCase inhibition or enhancement is reported relative to the control (without compounds). For each compound a blank composed by a water, compound and substrate (called “compound blank”) was tested and compared with the experiment blank, composed by water and substrate. No “compound blank” differs from the experiment blank, demonstrating that the compounds do not interfere with the fluorescence of the hydrolyzed substrate. Data are presented as mean ± standard deviation (n = 3).

The IC_50_ values of inhibitors against rh-GCase were determined by measuring the initial hydrolysis rate with 4-methylumbelliferyl-β-d-glucoside (3.33 mM). For more details see Supporting Information.

Kinetic analysis of compounds **1** and **2**: The action mechanism of compounds **1** and **2** was determined by studying the dependence of the main kinetic parameters (Km and Vmax) from the inhibitor concentration. Kinetic data were analyzed using the Lineweaver–Burk plots. For more details see Supporting Information.

### 2,2-Diphenyl-1-picrylhydrazyl (DPPH) Assay

The assay was performed according to a literature reported procedure.^45^ To 1.98 mL of a 200 μM DPPH solution in ethanol, 20 μL of a 20 mM ethanolic solution of compounds **1–3**, **8** and dibutylhydroxytoluene (BHT) were added and rapidly mixed. The progress of the reaction was monitored by spectrophotometric analysis measuring the absorbance at 515 nm every 30 s for 10 min. For more details see Supporting Information.

### Chaperoning Activity Assays on Gaucher-Derived Fibroblasts^32,47^

Gaucher disease patients’ cells were obtained from the “Cell line and DNA Biobank from patients affected by Genetic Diseases” (Gaslini Hospital, Genova, Italy)—Telethon Genetic Biobank Network). The cell passage number of fibroblasts is between 16 to 25.

To determine the GCase activity in intact cells, 3000–4000 cells were plated into 96-well black plate with clear bottom in D-MEM, 10% FBS at 37 °C under 5% CO_2_, either with or without the tested compounds in a dose-dependent manner at several concentrations. After 24 h of attachment, the medium was replaced with a fresh medium containing new compounds or Isofagomine (IFG) or Ambroxol (ABX) (as a control, they reached clinical trials for GD or/and GD/PD treatment). The cells were incubated for 4 days with the medium for DMSO-treated cells or compound-treated cells. The final concentration of DMSO was <0.5% which had no cytotoxicity. The enzyme activity assay was performed after removing the medium supplemented with compounds. The monolayers were washed with Dulbecco’s PBS solution (Gibco, pH 7.4). PBS (16 μL) and acetate buffer (0.2 M, 16 μL, pH 4.0) were added to each well. The reaction was started by the addition of 4-methylumbelliferyl β-d-glucoside (4 mM, 25 μL, Sigma) to each well, followed by incubation at 37 °C for 2 h. The reaction was stopped by lysing the cells with glycine buffer (0.2 M, 200 μL, pH 10.8). The fluorescence was also measured by the release of 4-methylumbelliferone with an excitation wavelength of 355 nm and an emission wavelength of 460 nm (SpectraMax M5, Molecular Devices). The fluorescence of untreated cells was compared with those treated with compounds. For the initial screening, treatments were evaluated in two independent biological experiments (n = 2), with untreated controls and compound-treated conditions analyzed in eight and five technical replicates, respectively. Selected compounds and concentrations were subsequently evaluated in three additional independent experiments, resulting in a total of five independent biological replicates (n = 5) for these conditions. Technical replicates from each independent experiment were averaged to obtain a single value for each biological replicate and were not considered as independent observations for statistical analysis. Under these experimental conditions, the activity of nonlysosomal β-glucosidase was shown to be negligible (typically around 5%) at pH 4. This was confirmed by J. W. Kelly and coworkers using conduritol B epoxide (CBE), an irreversible inhibitor that specifically targets lysosomal GCase but does not affect nonlysosomal β-glucosidase. As expected, the CBE-insensitive activity was minimal, indicating that the observed GCase activity largely originated from the lysosomal enzyme.

Statistical Analysis: Statistical significance was evaluated using Student’s *t* test. A p-value lower than 0.05 was considered to be statistically significant. The single (*), double (**), triple (***) and quadruple (****) asterisks refer to p-values lower than 0.05, 0.01, 0.001 and 0.0001, respectively. Statistical analysis was performed using the GraphPad Prism 8.

### Flow Cytometric Analysis of ROS Levels in Cells Treated with Compound **2** ^32^

Fibroblasts derived from GD patients bearing L444P/L444P were seeded in P60 culture dishes and maintained at 37 °C in a controlled atmosphere with 5% CO_2_. Once cells reached confluence, the growth medium was replaced with fresh medium containing either 1 μM of compound **2** or vehicle, and cells were incubated for an additional 24 h at 37 °C. After incubation, the medium was removed, and cells were washed with PBS, trypsinized, and collected into 15 mL conical Falcon tubes. Cells were centrifuged (5 min at 1000 rpm), then washed with 1X wash buffer provided in the ROS-ID Total ROS/Superoxide Detection Kit (Enzo Life Sciences, Enzo Biochem, Inc., 21 Executive Blvd., Farmingdale, NY 11735). The resulting cell pellet was resuspended in 500 μL of ROS detection solution included in the kit and incubated at 37 °C for 30 min. Finally, cells were centrifuged and resuspended in 120 μL of PBS prior to analysis by flow cytometry (FACSCanto II, BD Biosciences).

### Pharmacological Chaperoning Activity in Wild-Type Fibroblasts^50^

Wild-type fibroblasts were obtained from the “Cell Line and DNA Biobank from Patients Affected by Genetic Diseases” at Gaslini Hospital (Genoa, Italy). Cells (1.35–1.50 × 10^5^) were seeded in T25 culture flasks containing DMEM supplemented with 10% fetal bovine serum, 1% penicillin/streptomycin, and 1% glutamine, and incubated for 24 h at 37 °C in a humidified atmosphere with 5% CO_2_. The culture medium was then replaced with fresh medium containing the tested compounds **1** and **2**, and cells were incubated for an additional 4 days. Following treatment, the medium was removed, and cells were washed with PBS and detached using trypsin. Cell pellets were collected, washed four times with PBS, frozen, and lysed by sonication in water. Total protein concentration in fibroblasts homogenates was determined using the Micro BCA Protein Assay Kit (Sigma–Aldrich), following the manufacturer’s instructions. GCase activity was measured in flat-bottom 96-well microplates in triplicates. The reaction mixture consisted of fibroblast homogenate (10 μL, 3 μg protein/μL), and 4-methylumbelliferyl-β-d-glucoside (20 μL, 3.33 mM; Sigma–Aldrich) dissolved in citrate/phosphate buffer (0.1/0.2 M, pH 5.8) containing sodium taurocholate (0.3%) and Triton X-100 (0.15%). Samples were incubated at 37 °C for 1 h. The reaction was terminated by adding 200 μL of sodium carbonate solution (0.5 M, pH 10.7) containing Triton X-100 (0.0025%). The fluorescence of the released 4-methylumbelliferone, generated by β-glucosidase activity, was measured using a SpectraMax M2 microplate reader (Molecular Devices) at excitation and emission wavelengths of 365 and 435 nm, respectively. Data are reported as mean ± SD from two technical replicates (n = 2).

## Supplementary Material



## Notes

The NMR spectra of compound **8** and the newly synthesized hybrids **2** and **3**, as well as the raw data from the biological (*in vitro* and *ex vivo*) studies (PDF)

## Abbreviations

ROS: reactive oxygen species
RNS: reactive nitrogen species
GD: Gaucher disease
GCase: β-glucocerebrosidase
PCs: pharmacological chaperones
ER: endoplasmic reticulum
PD: Parkinson’s disease
WT: wild-type
rh-GCase: recombinant human GCase
CuAAC: copper-catalyzed azide–alkyne cycloaddition
