# Supplementary material for: A pH-Dependent Curcumin–Iminosugar Hybrid Promotes GCase Enhancement and Reactive Oxygen Species Reduction
Source: ACS Omega. 2026 Oct 1;11(40):62107–14. doi: 10.1021/acsomega.6c08401 (PMC13670301; doi:10.1021/acsomega.6c08401)
Supplement: Supplementary file 1 [file ao6c08401_si_001.pdf]

Supporting Information

A pH-Dependent Curcumin–Iminosugar Hybrid Promotes GCase Enhancement and Reactive Oxygen Species Reduction.

Alessio Morano,[a] Camilla Matassini,[a] Francesca Cardona,*[a] Damiano Tanini,[a] Silvia Falliano,[b] Amelia Morrone,[c,b,] Rebecca Sodano,[d] Paolo Paoli,[d] Andrea Goti,[a] Francesca Clemente*[a]

[a] Department of Chemistry "Ugo Schiff" (DICUS), University of Florence, Via della Lastruccia 3–13, 50019 Sesto F.no (FI) (Italy);

[b] Laboratory of Molecular Genetics of Neurometabolic Diseases, Department of Neuroscience and Medical Genetics, Meyer Children's Hospital IRCCS, Viale Pieraccini 24,50139, Firenze (Italy);

[c] Department of Neurosciences, Psychology, Drug Research and Child Health (NEUROFARBA), University of Florence, Viale Pieraccini 24, 50139 Firenze (Italy);

[d] Department of Experimental and Clinical Biomedical Sciences (DSBSC), University of Florence, Viale Morgagni 50, 50134 Firenze (Italy).

Table of contents

NMR spectra of compounds **8**, **2** and **3**	S3
Activity towards *rh*-GCase	S6
Kinetic analysis of compounds **1** e **2**	S12
2,2-Diphenyl-1-picrylhydrazyl (DPPH) assay	S15
Chaperoning activity assays in Gaucher-derived fibroblasts	S16
Flow cytometric analysis of ROS levels in cells treated with compound **2**	S22
Chaperoning activity assays in wild-type fibroblasts	S23

(3*R*,5*R*)-1-Octyl-3,4,5-trihydroxy piperidine (8)

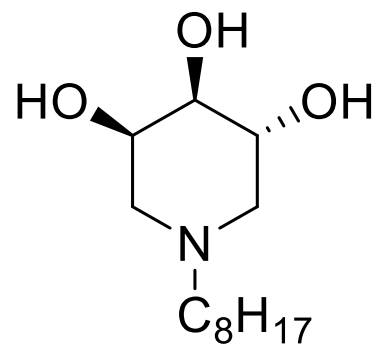

8

$C_{13}H_{27}NO_3$ (245.20): MS (ESI): *m/z* (%) = 246.66 (100) $[M+H]^+$.

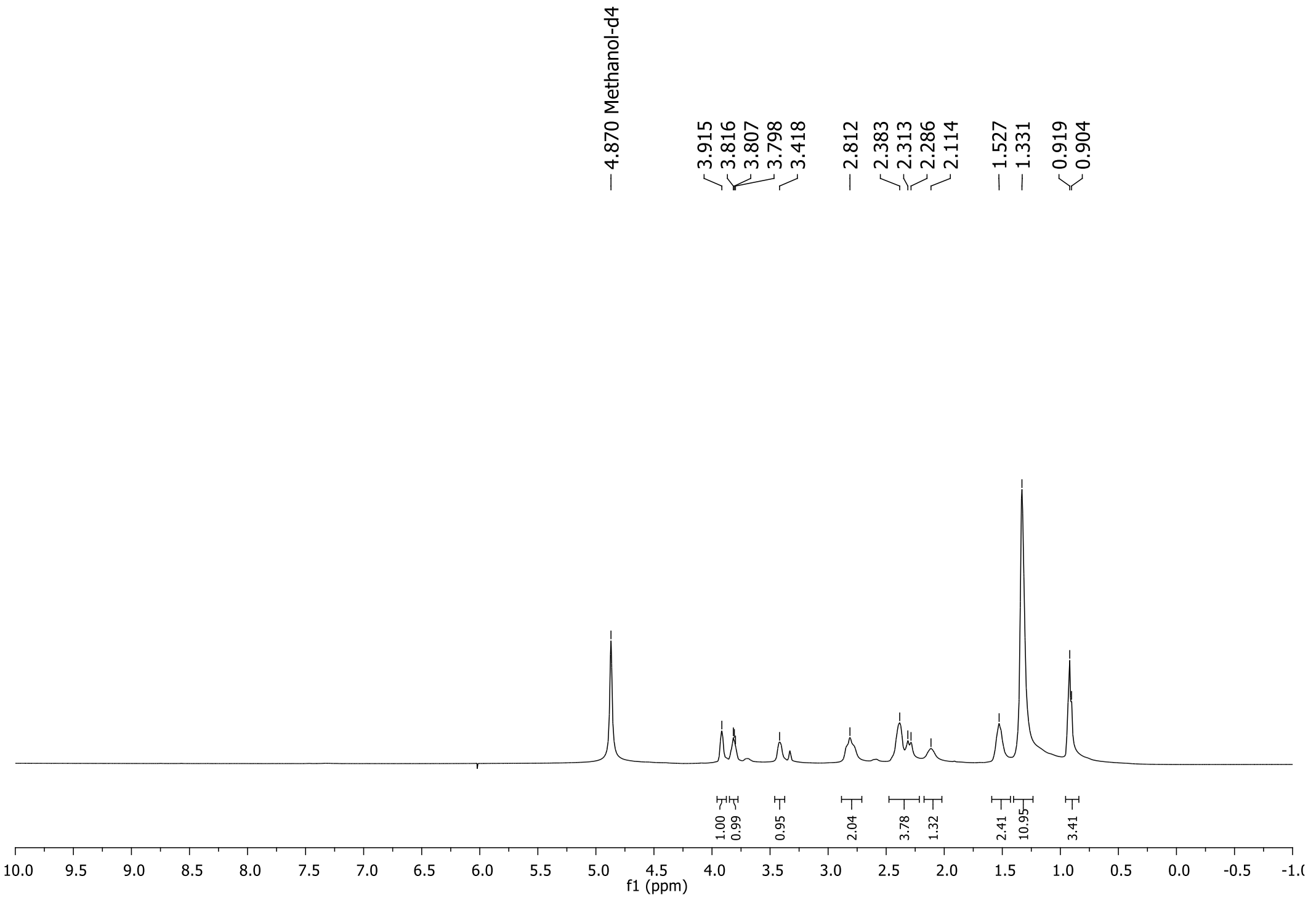

Figure S1: ^{1}H-NMR spectrum of compound **8** (CD_3OD, 400 MHz).

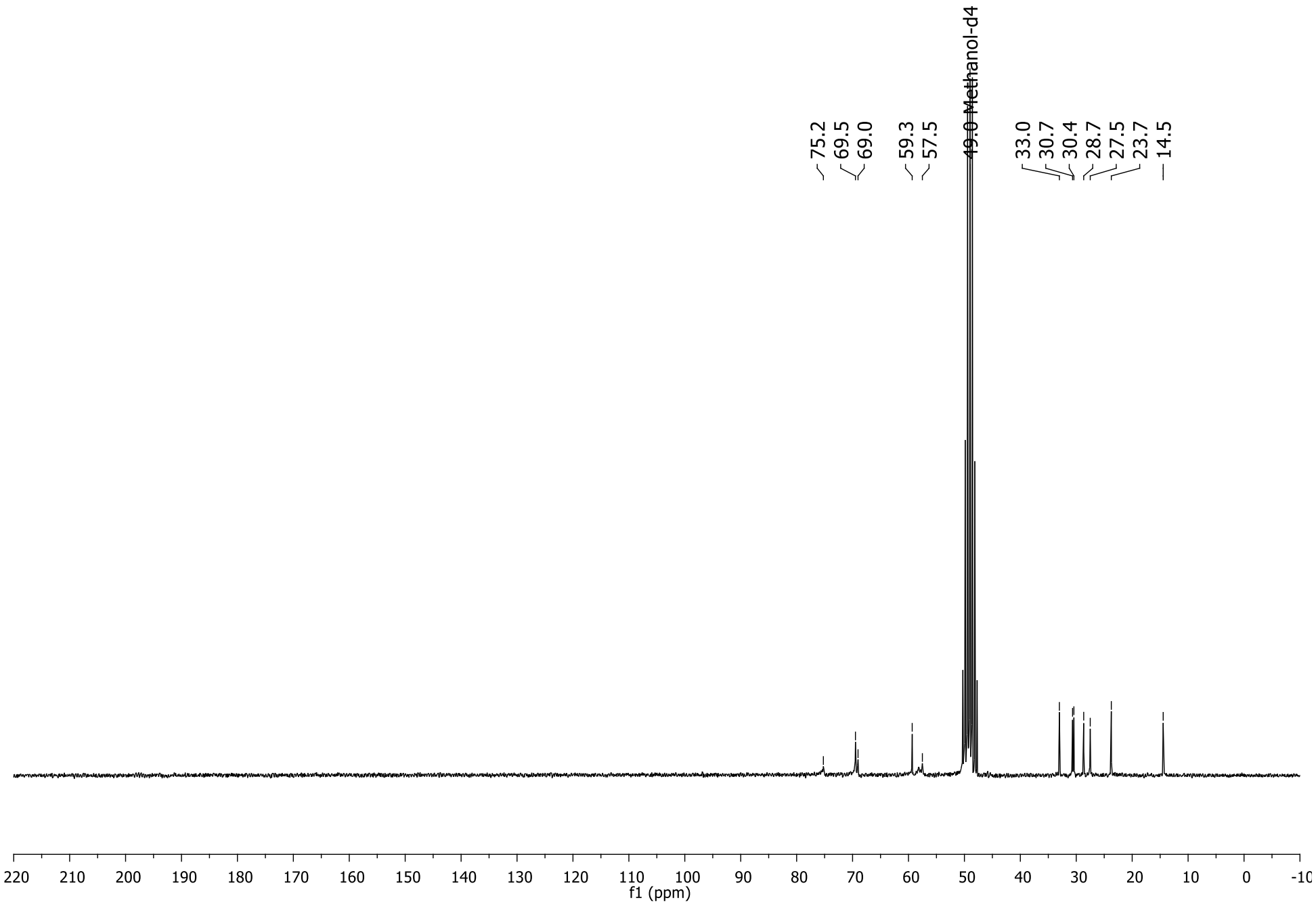

Figure S2: ^{13}C-NMR spectrum of compound **8** (CD_3OD, 100 MHz).

NMR spectra of compounds 2 and 3

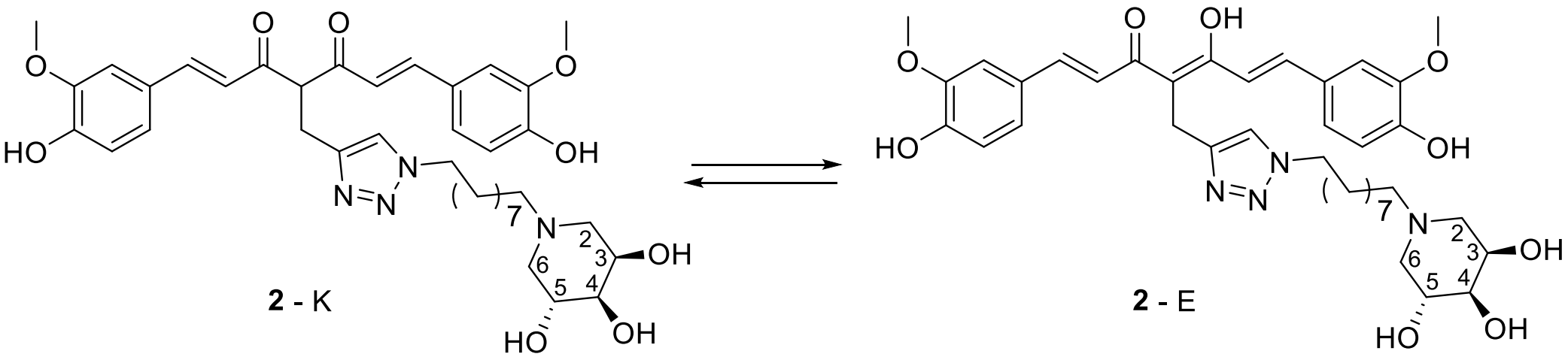

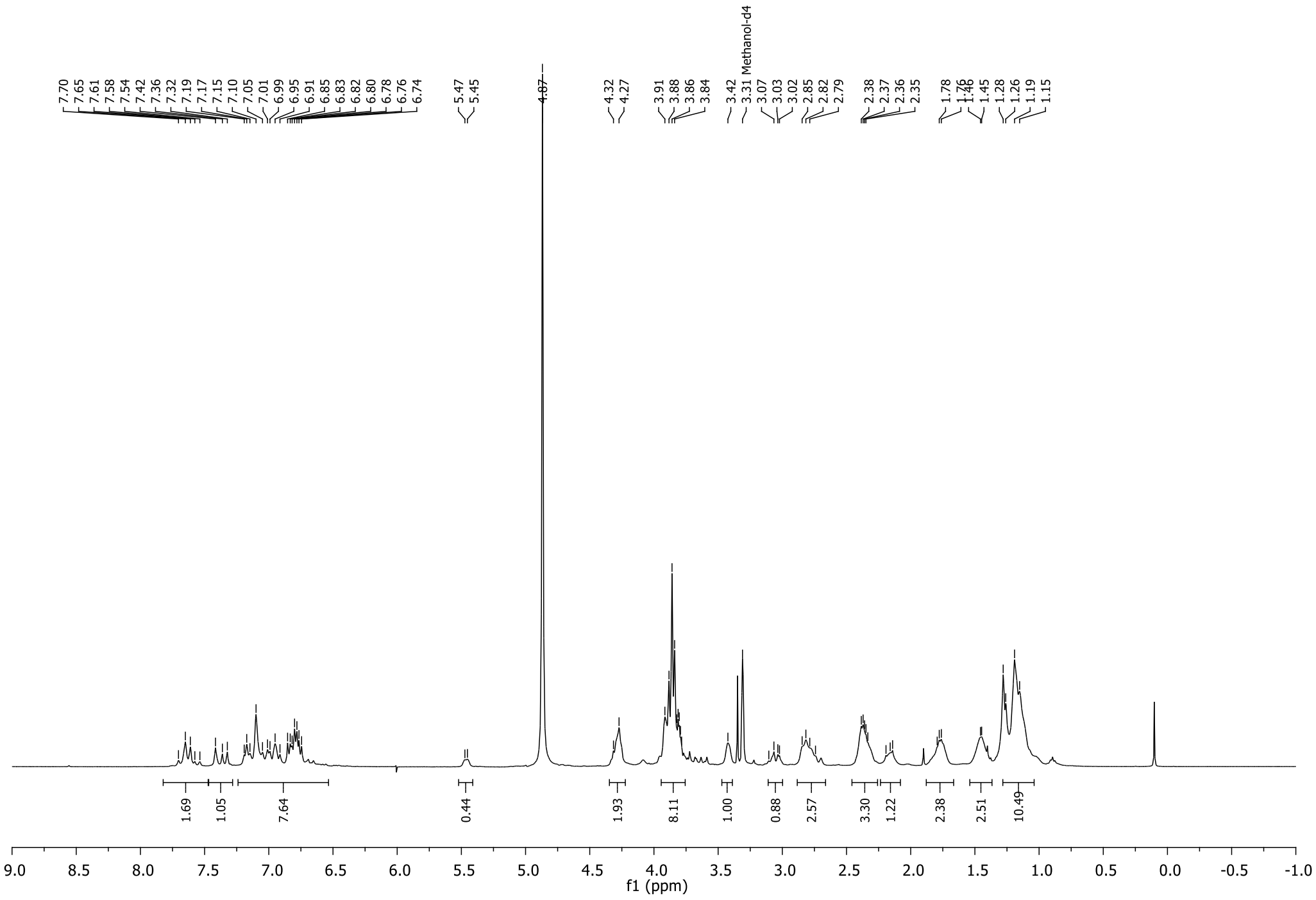

Figure S3: ^{1}H-NMR spectrum of compound **2** (CD_3OD, 400 MHz).

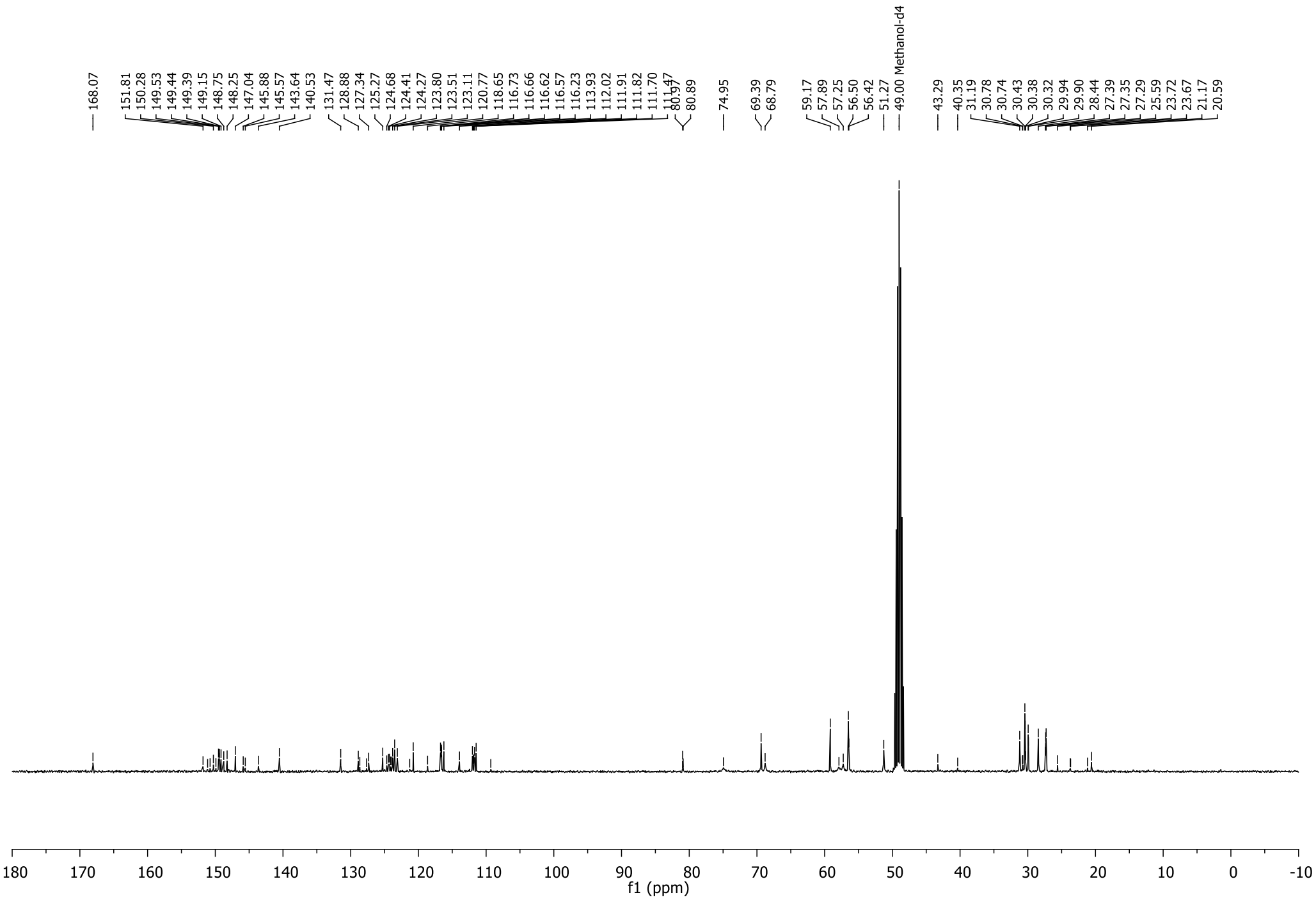

Figure S4: ^{13}C-NMR spectrum of compound **2** (CD_3OD, 100 MHz).

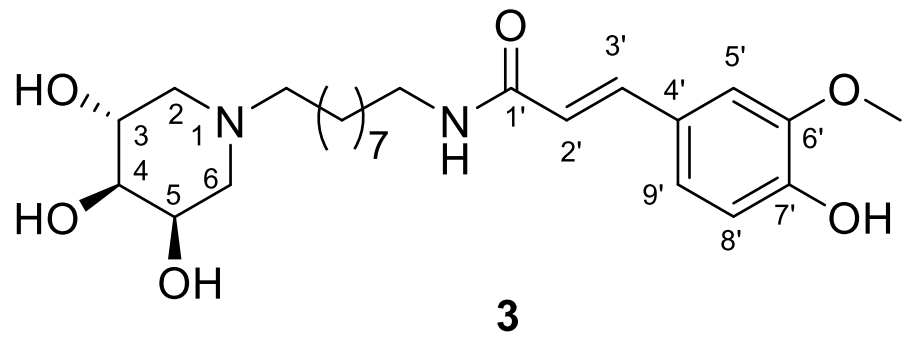

3

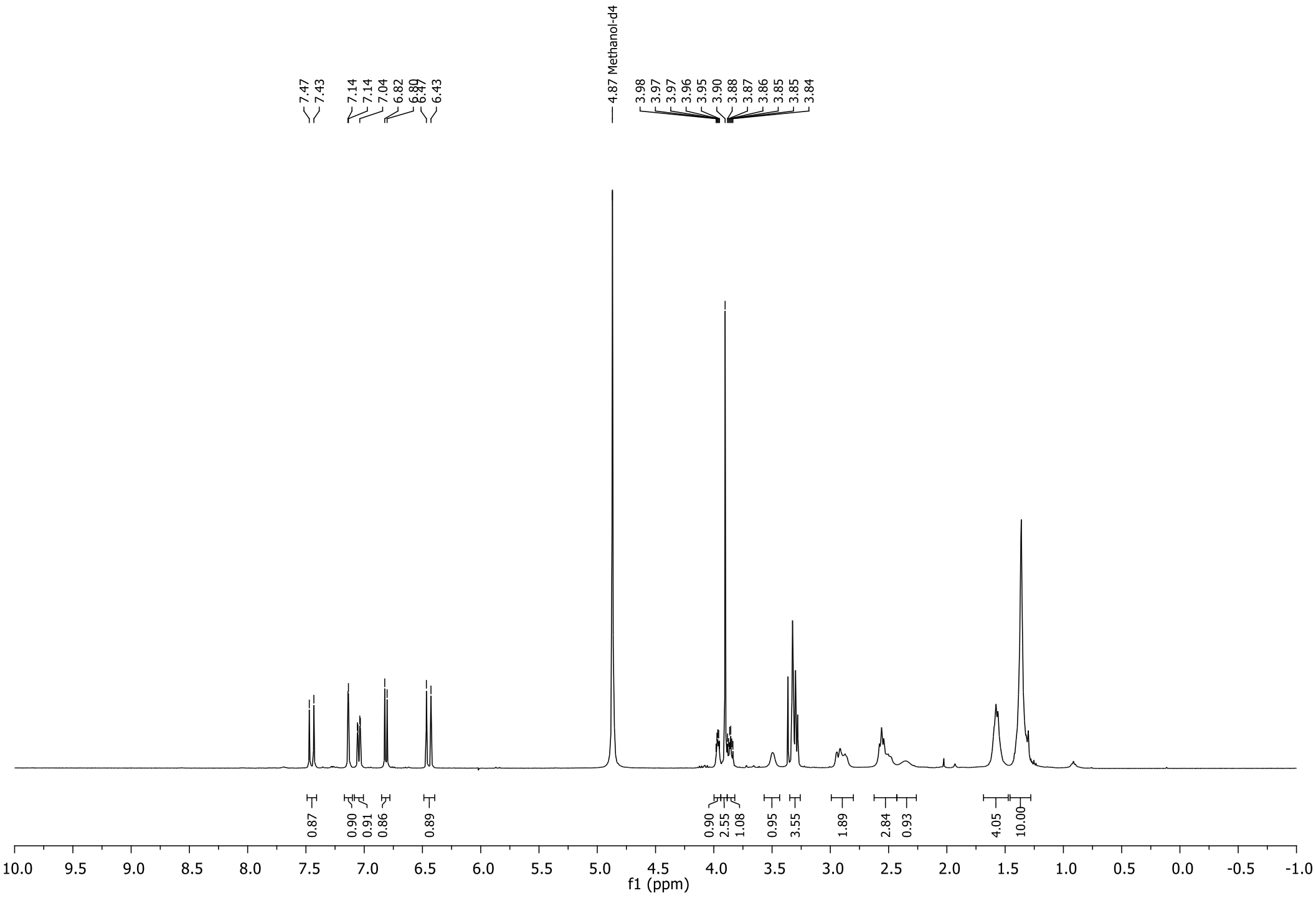

Figure S5: ^{1}H-NMR spectrum of compound **3** (CD_3OD, 400 MHz).

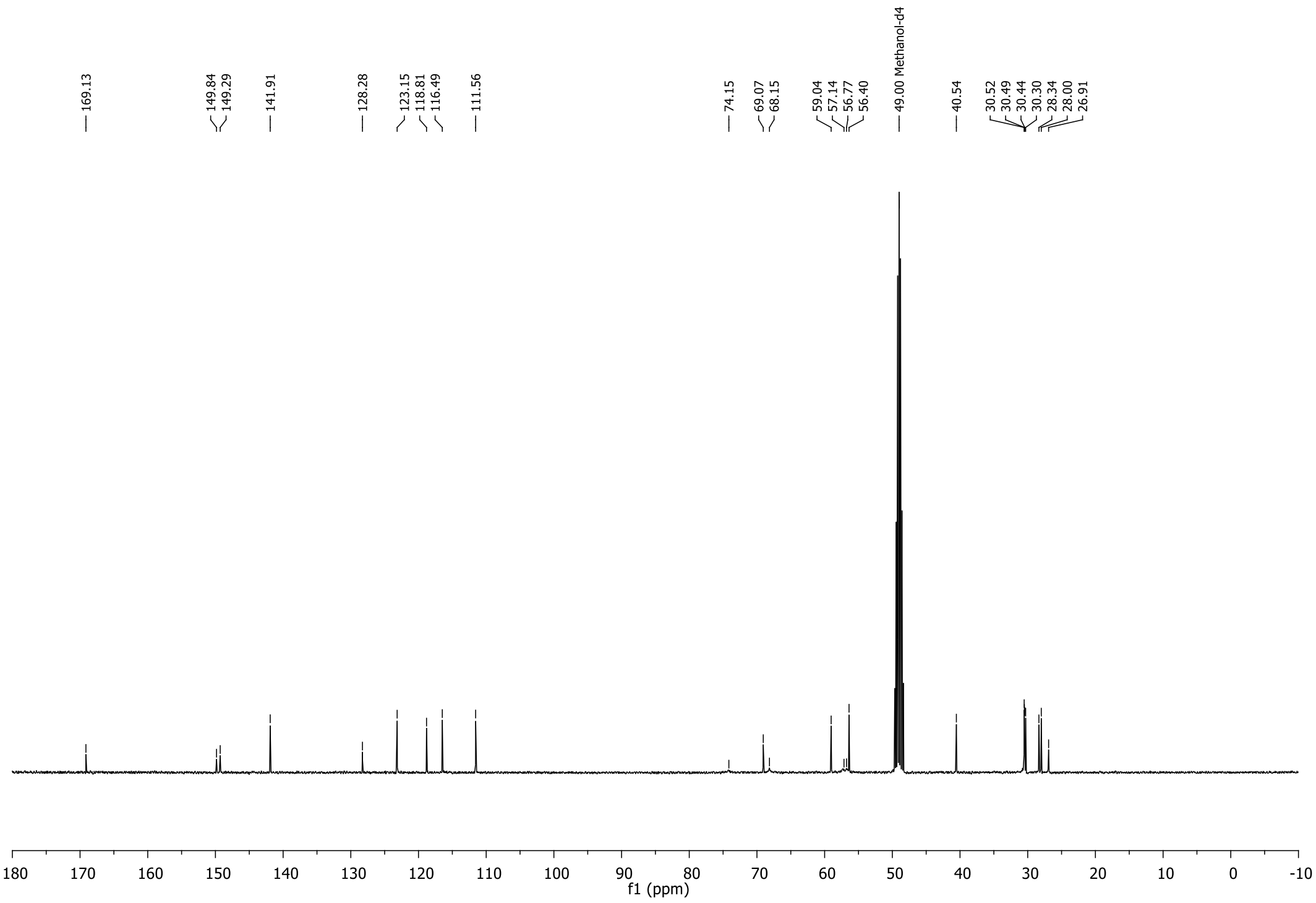

Figure S6: ^{13}C-NMR spectrum of compound **3** (CD_3OD, 100 MHz).

Activity towards human GCase

The IC_{50} values of inhibitors against *rh*-GCase were determined by measuring the initial hydrolysis rate with 4-methylumbelliferyl-β-D-glucoside (3.33 mM). Data obtained were fitted to the following equation using the Origin Microcal program.

$$\frac{Vi}{Vo} = \frac{Max - Min}{1 + \left(\frac{x}{IC_{50}}\right)^{slope}} + Min$$

rh-GCase activity at pH 7.0

IC_{50}= 12.0 ± 2.4 μM

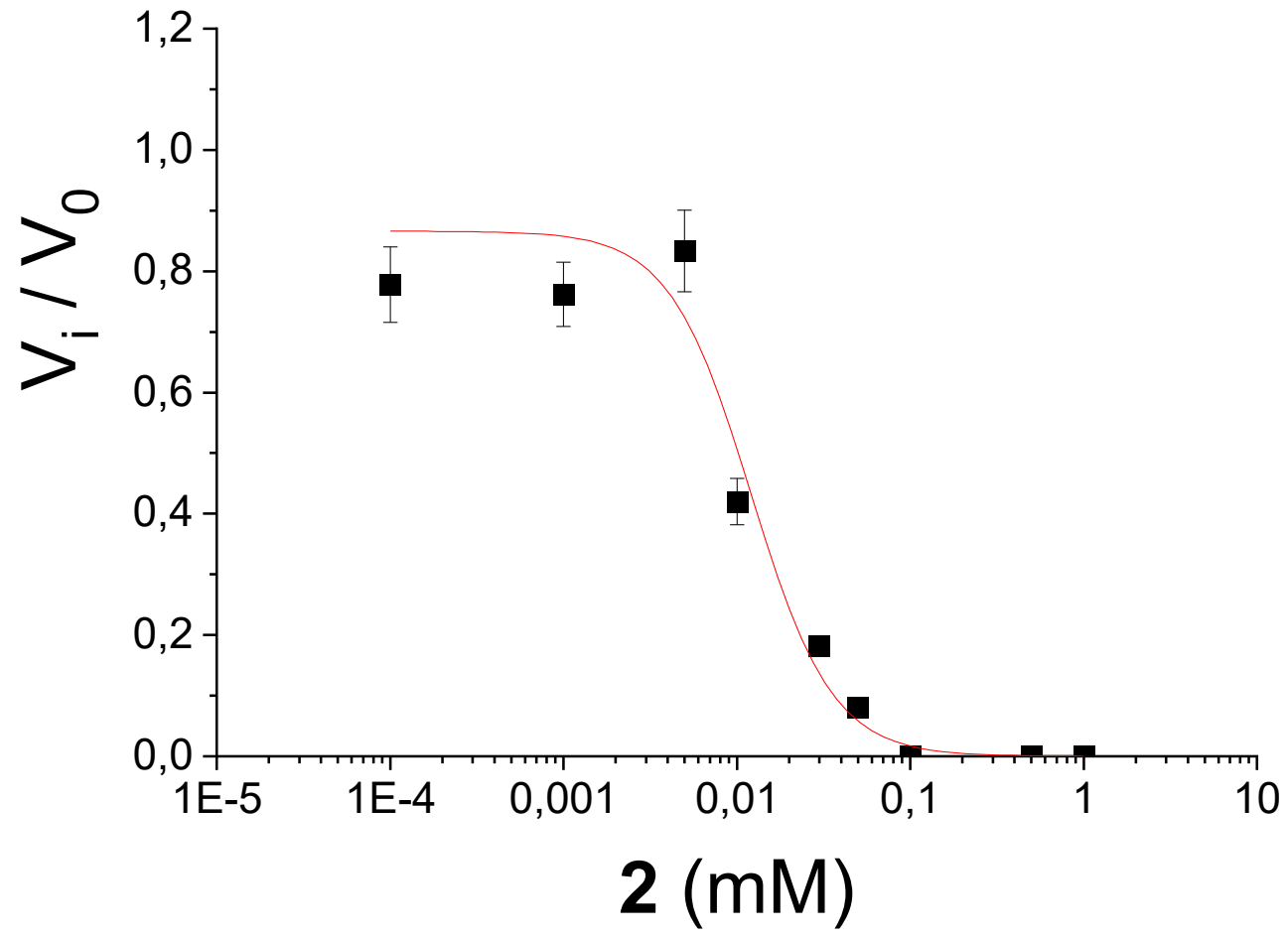

Figure S7: IC_{50} graph of compound **2**

IC_{50}= 20 ± 1 µM

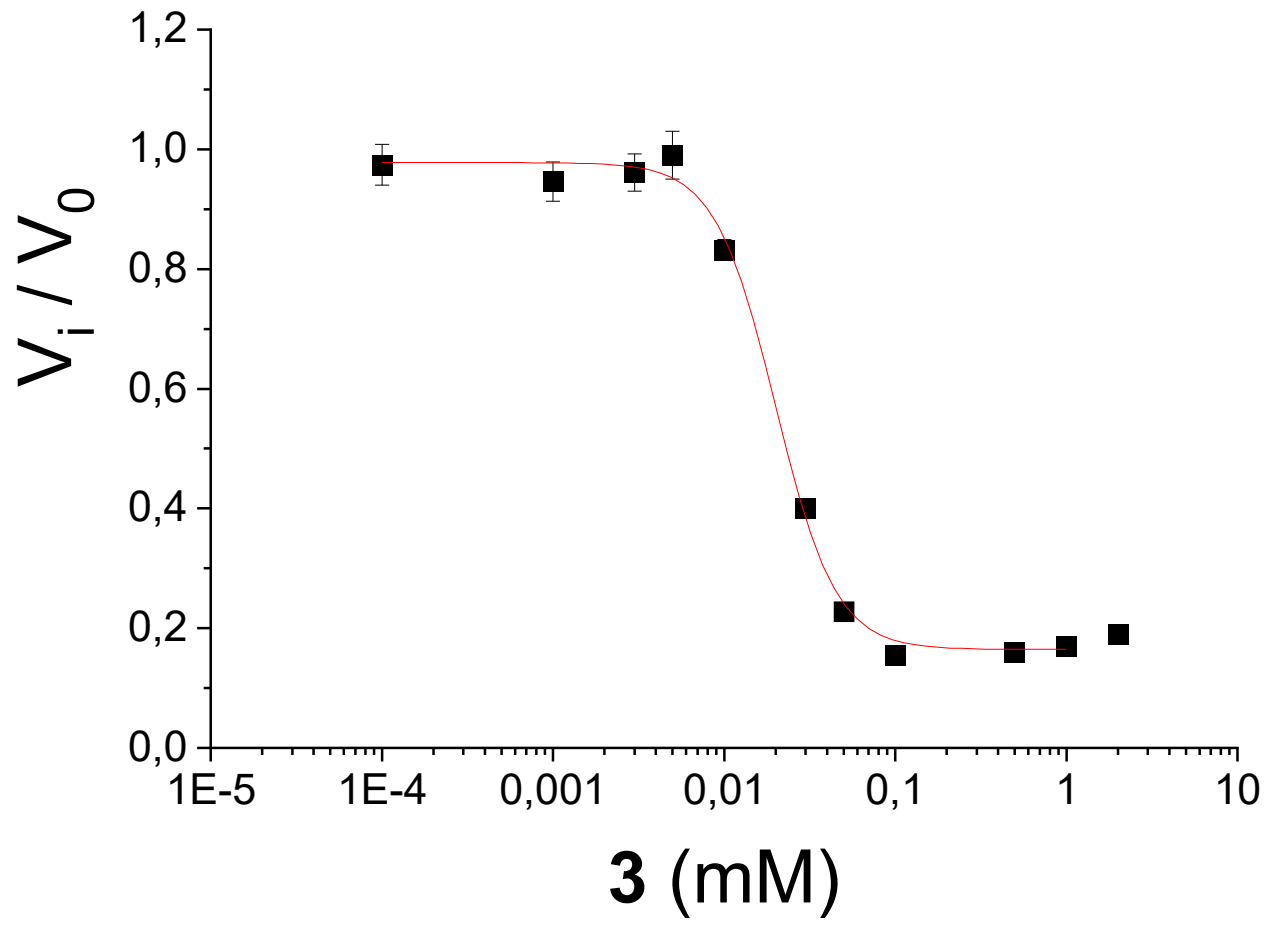

Figure S8: IC_{50} graph of compound **3**

IC_{50} = 21.0 ± 3.3 µM

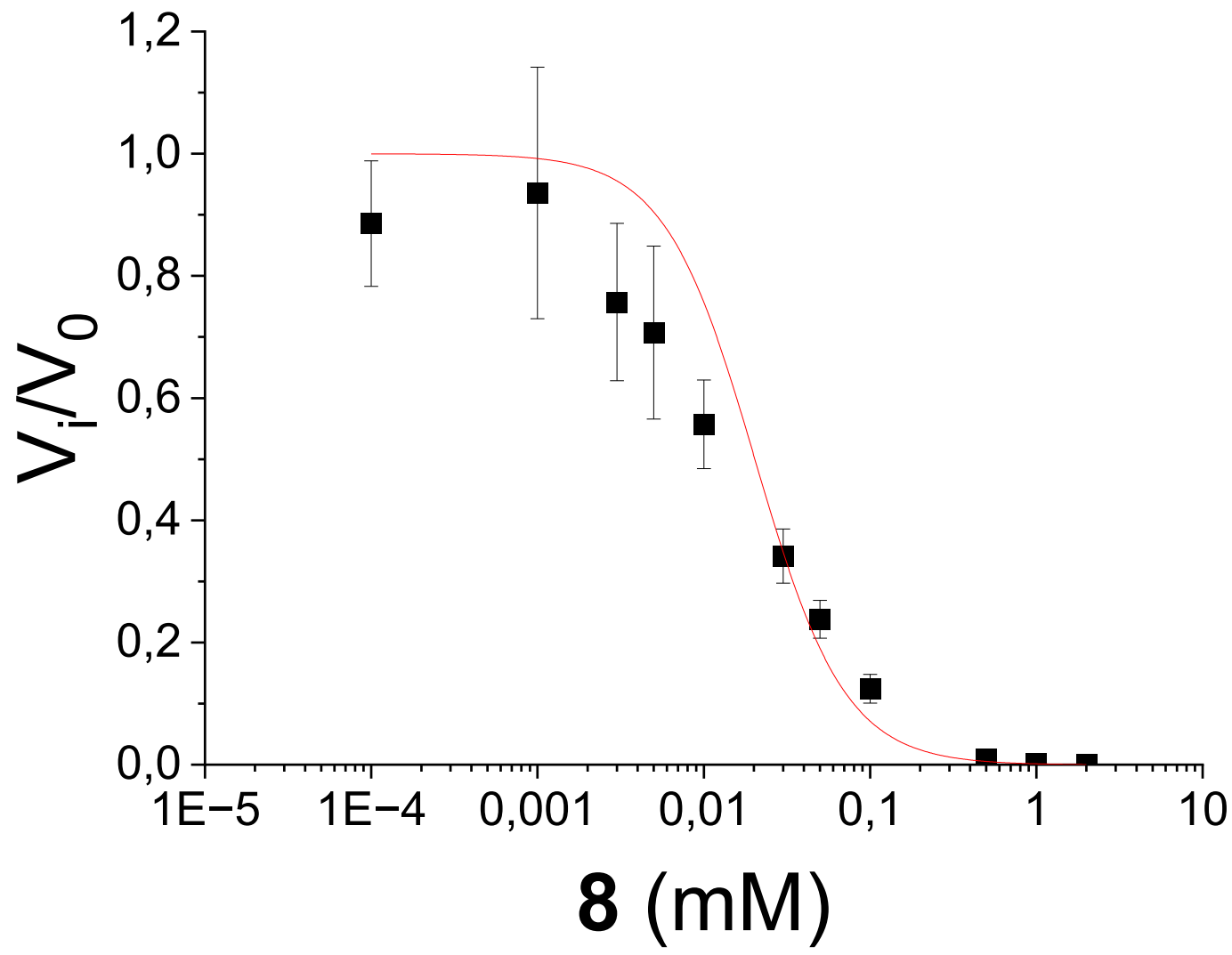

Figure S9: IC_{50} graph of compound **8**

IC50 ≈ 500 µM

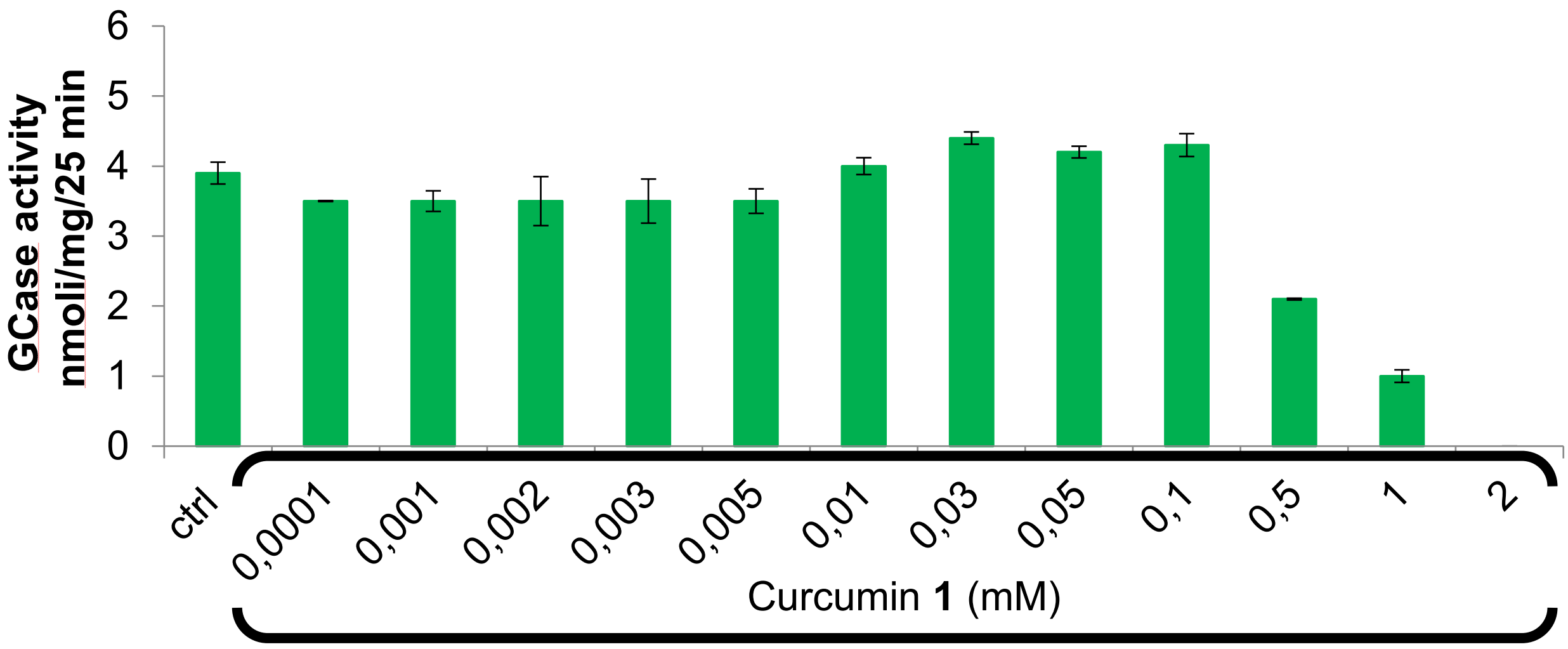

Figure S10: Activity of recombinant human GCase (*rh*-GCase) in the absence (ctrl) and presence of Curcumin (**1**). Data are presented as mean ± standard deviation (n = 3).

***rh*-GCase activity at pH 4.5**

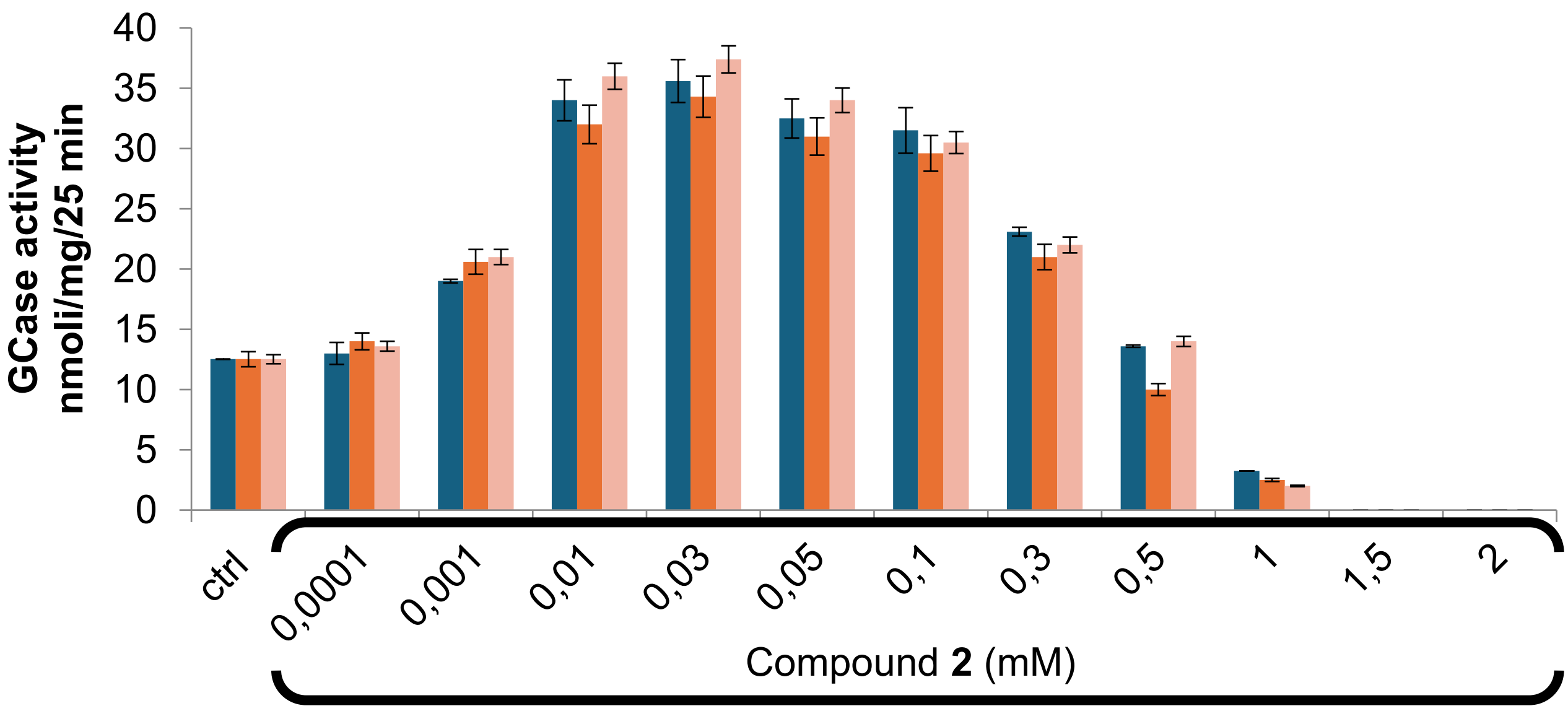

Figure S11: Activity of recombinant human GCase (*rh*-GCase) in the absence (ctrl) and presence of compound **2**. Data are presented as mean ± standard deviation (n = 3). Different colors represent independent experiments.

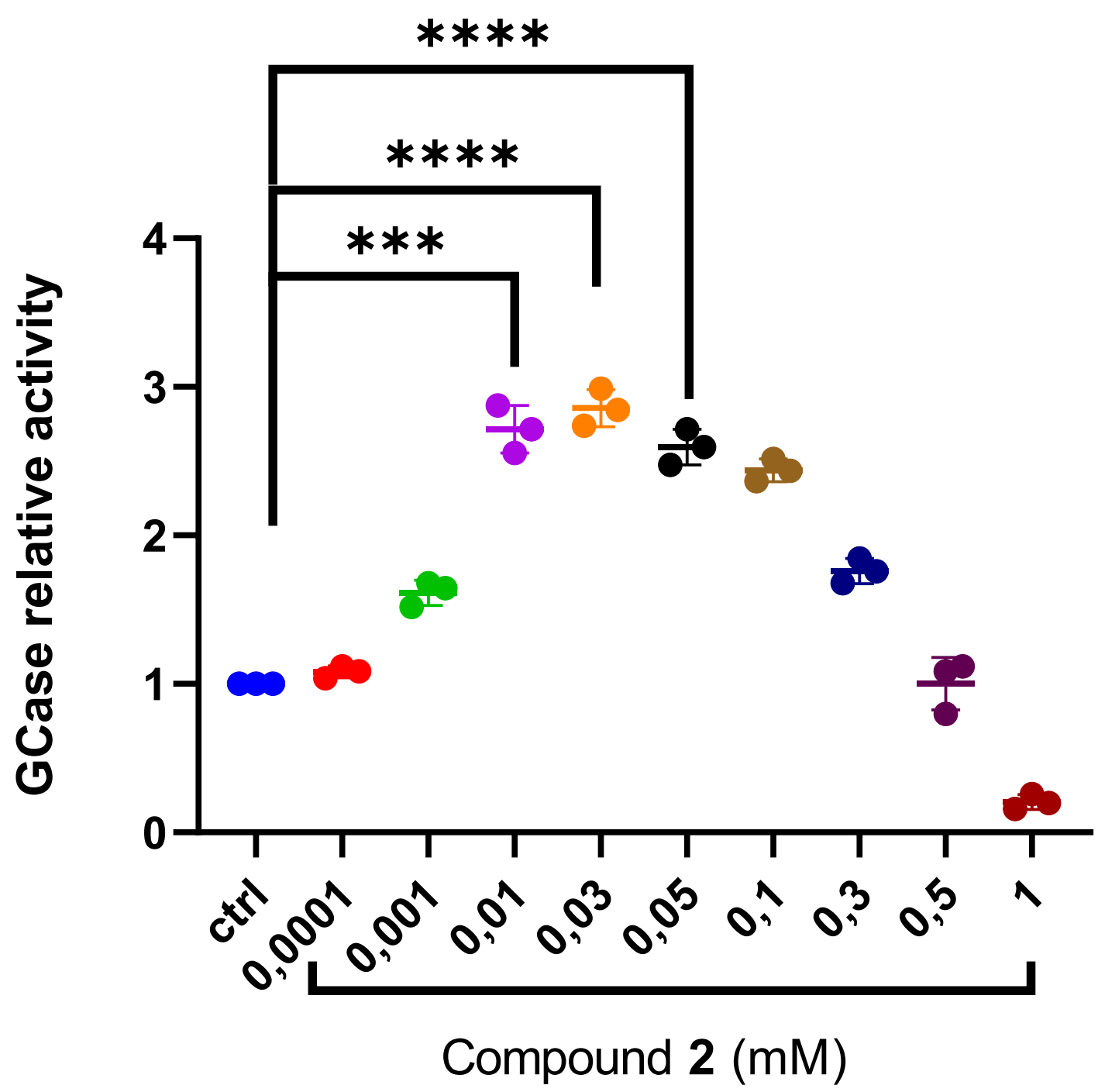

Figure S12: Relative GCase activity is expressed as the activity with the compound divided by the activity in its absence (ctrl).Statistical significance was determined using Student's t-test: * p ≤0.05, ** p ≤ 0.01, *** p ≤ 0.001, and **** p ≤ 0.0001. Each data point represents the analysis of triplicates for both the control group and the samples, based on N = 3 independent experiments.

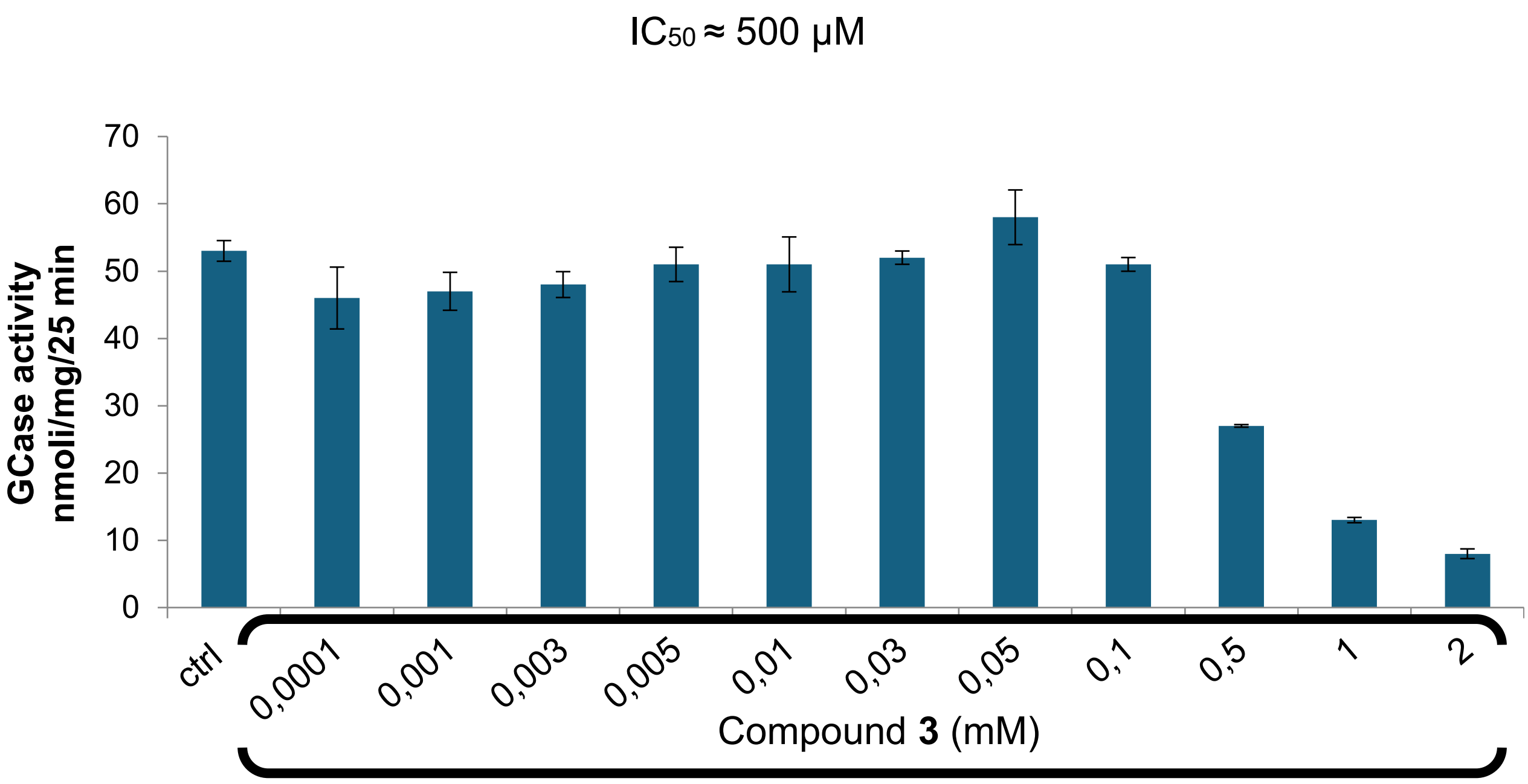

Figure S13: Activity of recombinant human GCase (*rh*-GCase) in the absence (ctrl) and presence of compound **3**. Data are presented as mean ± standard deviation (n = 3).

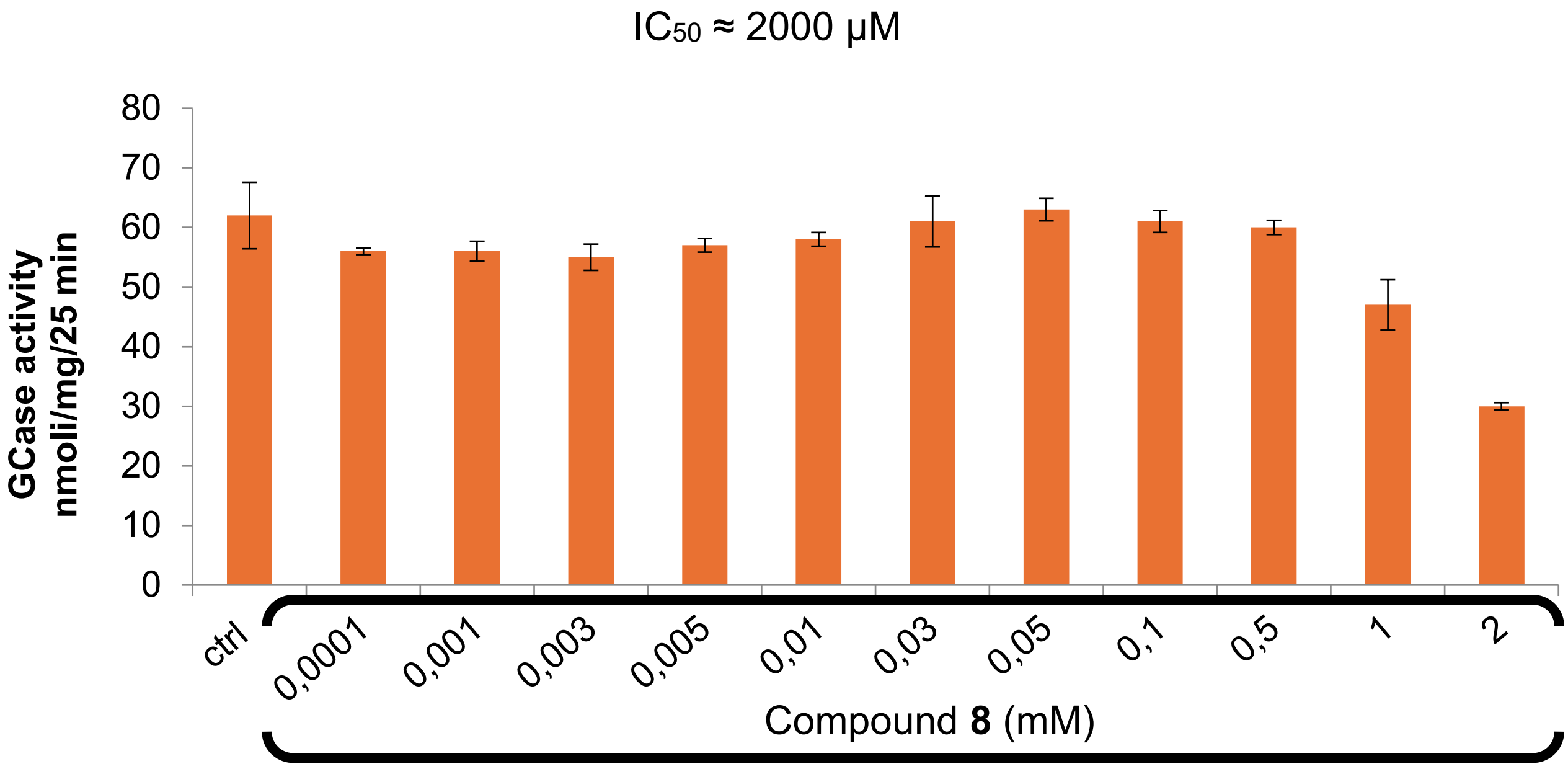

Figure S14: Activity of recombinant human GCase (*rh*-GCase) in the absence (ctrl) and presence of compound **8**. Data are presented as mean ± standard deviation (n = 3).

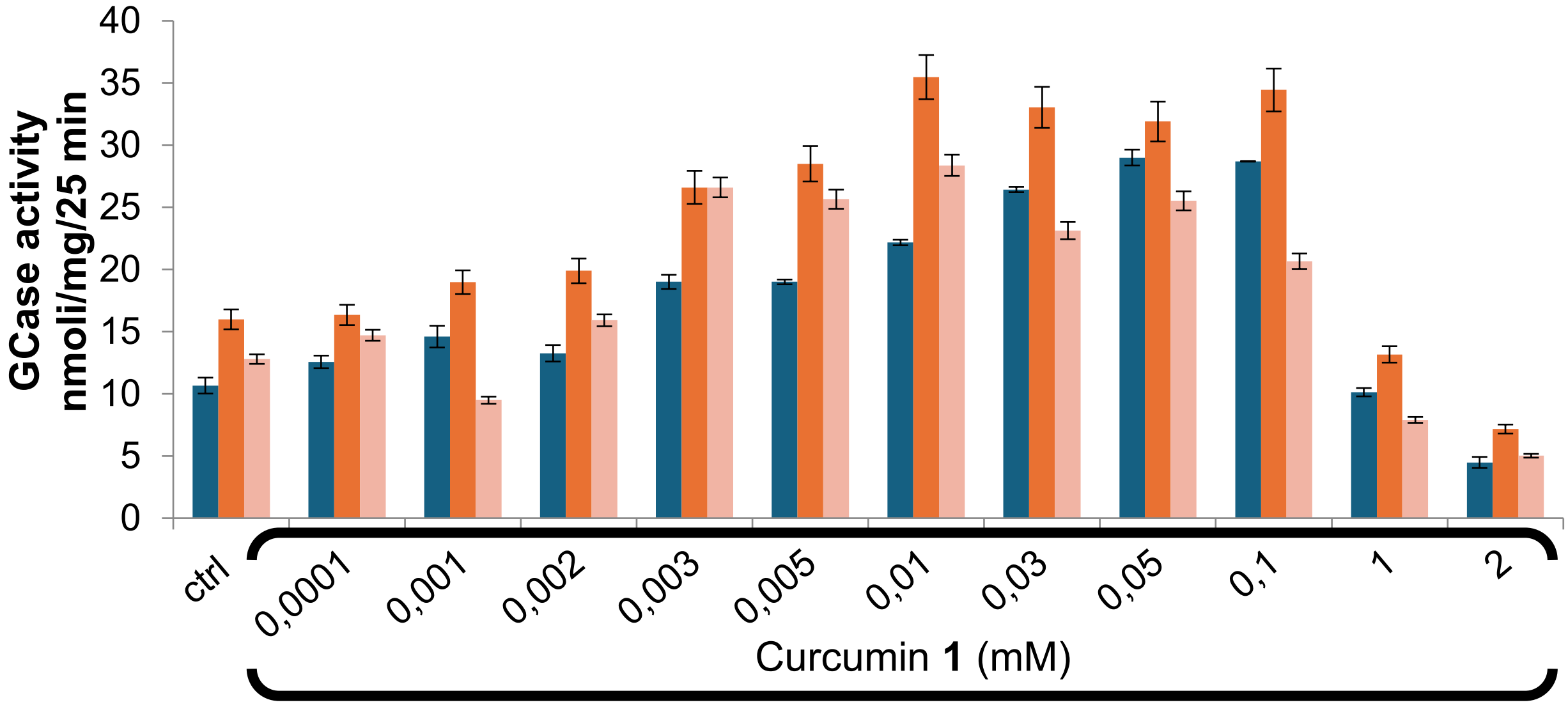

Figure S15: Activity of recombinant human GCase (*rh*-GCase) in the absence (ctrl) and presence of Curcumin (**1**). Data are presented as mean ± standard deviation (n = 3). Different colors represent independent experiments.

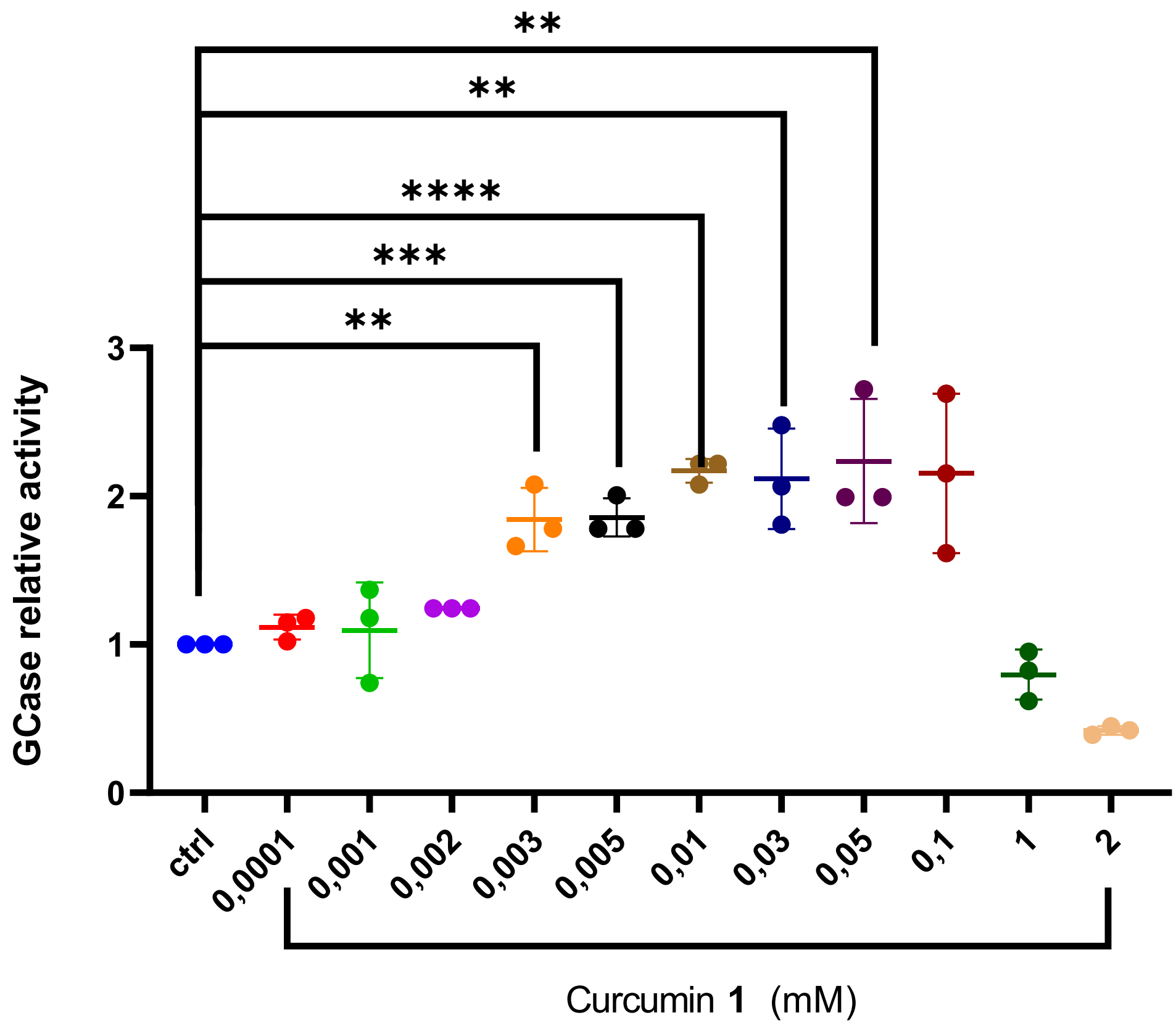

Figure S16: Relative GCase activity is expressed as the activity with the compound divided by the activity in its absence (ctrl).Statistical significance was determined using Student's t-test: * p ≤0.05, ** p ≤ 0.01, *** p ≤ 0.001, and **** p ≤ 0.0001. Each data point represents the analysis of triplicates for both the control group and the samples, based on N = 3 independent experiments.

Kinetic analysis of compounds 1 e 2

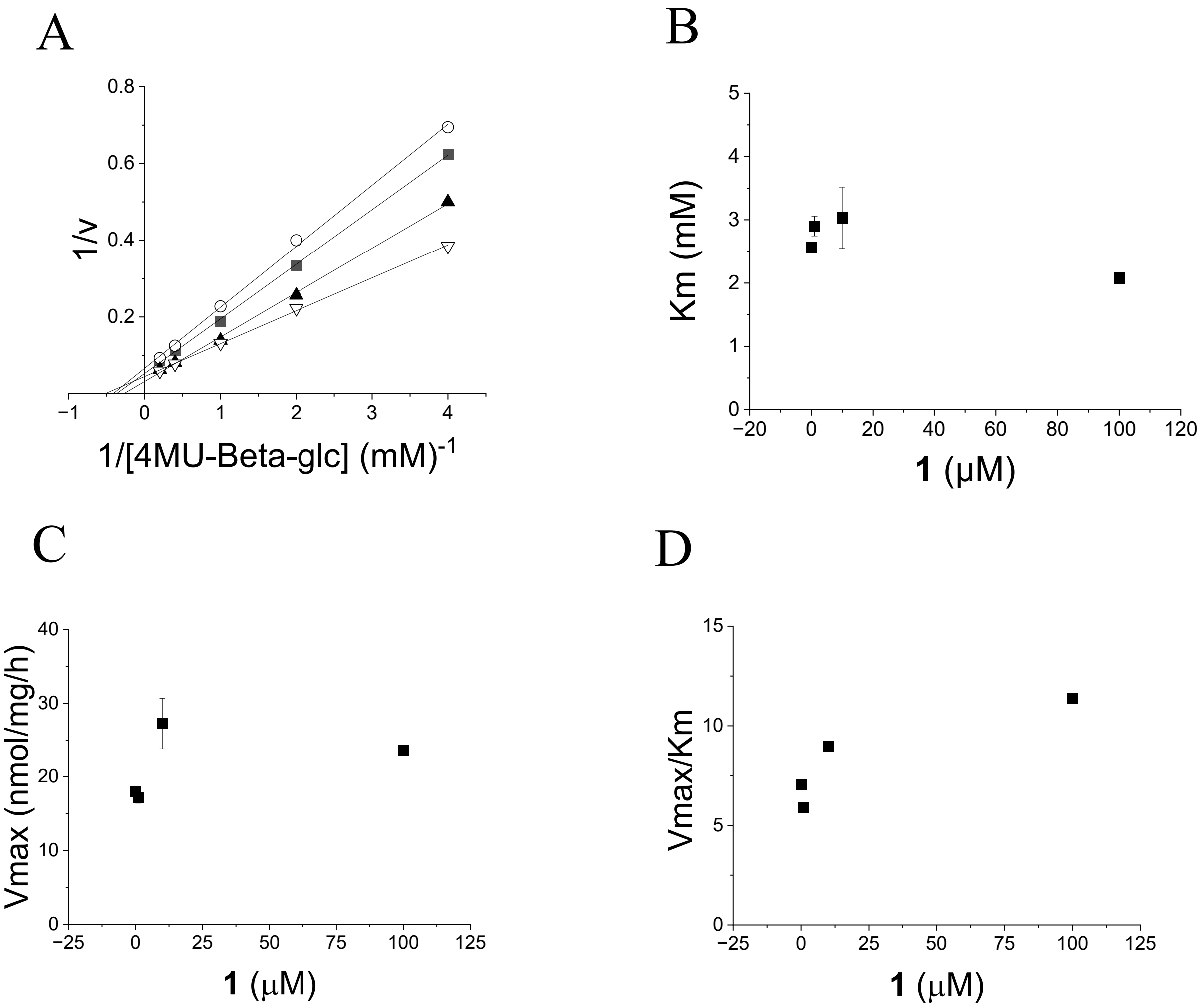

Figure S17: kinetic analysis of Curcumin (**1**). A), Lineweaver-Burk plot. Concentrations of **1** used were: 0 (■), 1 (○), 10 (▲) and 100 (▽) μM. (B-D) Dependence of Km, Vmax and Vmax/Km on **1** concentration.

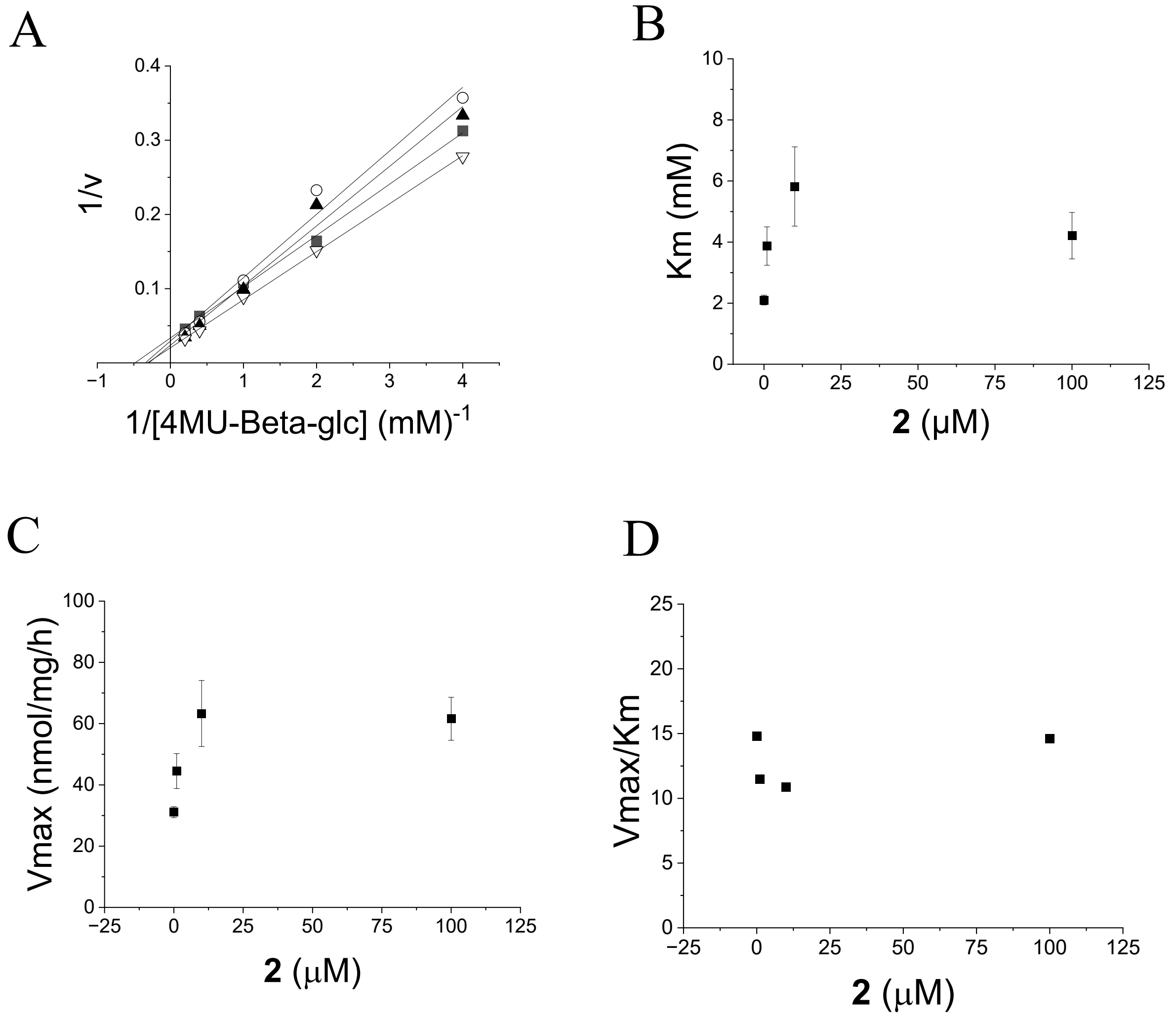

Figure S18: mechanism of action of compound **2**. (A), Lineweaver-Burk plot. Concentrations of **2** used were: 0 (■), 1 (○), 10 (▲) and 100 (▽) μM. (B-D) Analysis of dependence of the main kinetic parameters (Km and Vmax) or Vmax/Km ratio from compound **2**.

To gain insight into the mechanism of action of compound **1**, kinetic data were analysed using Lineweaver–Burk plots (Fig. S17A). The experimental data yield straight lines intersecting in the left quadrant, consistent with non-competitive behaviour. Accordingly, **1** has only a minor effect on Km, while exerting a pronounced effect on Vmax of GCase, indicating that its activity arises from binding to a site distinct from the catalytic centre. Although at 1 μM compound **1** induces a modest increase in Km (Fig. S17B) without affecting Vmax (Fig. S17C), at higher concentrations it behaves as a **non-essential activator**, as evidenced by a marked increase in Vmax while substrate affinity remains unchanged or is reduced relative to the control. This interpretation is further supported by the progressive increase in the Vmax/Km ratio at 10 and 100 μM of compound **1** (Fig. S17D). Therefore, the mechanism of action of compound **1** can be summarized as shown in Figure S19. The data in Fig. S17 show that the activator only slightly affects Km over the 1–100 μM range (Fig. S17B), indicating that the value of parameter "**α**" approaches unity. In contrast, the increase in Vmax with increasing **1** concentration indicates that β > 1. As [A] increases

from zero to infinity, $V_{max,app}$ increases from Vmax to βVmax. Then, the β value can derived easily from the βVmax / Vmax. In this specific case, the β value resulted to be 1.32.

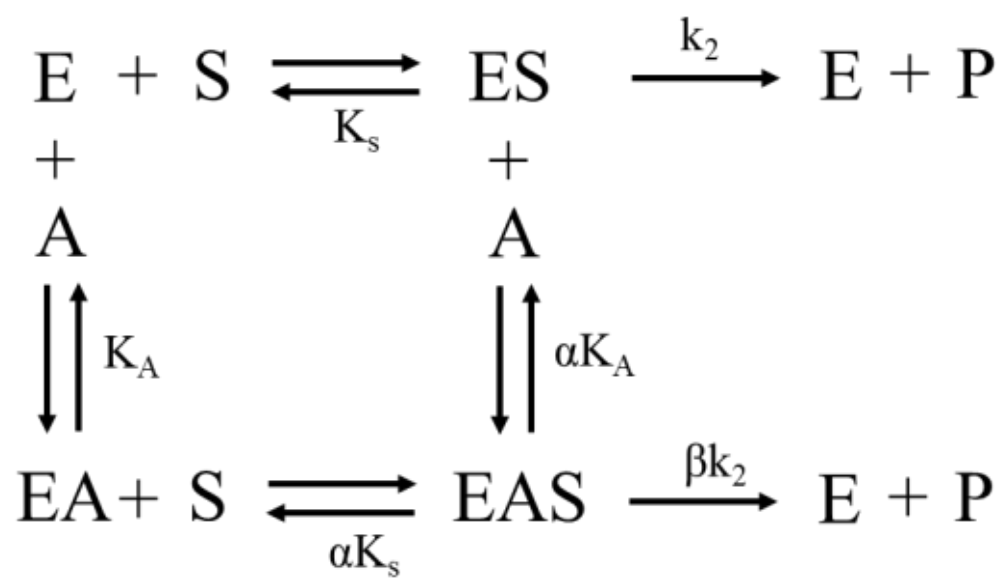

Figure S19: scheme of non-essential activation where E, ES, EA, EAS and P denote the free enzyme, the enzyme–substrate complex, the enzyme–activator complex, the enzyme-activator-substrate ternary complex and the reaction product, respectively. Moreover, K_s and K_a represent the dissociation constants for the enzyme–substrate and enzyme–activator interactions.

Kinetic analyses of compound **2** (Fig. S18) indicate that it only partially recapitulates the behaviour of **1**. Lineweaver–Burk plots (Fig. S18A) show that the fitted lines intersect the 1/[S] axis at distinct points relative to the control, indicating a pronounced effect on Km. Accordingly, Km increases sharply at 1 μM **2**, with little further change at higher concentrations (Fig. S18B). In parallel, compound **2** enhances GCase activity, as reflected by an increase in Vmax (Fig. S18C), consistent with non-essential activation. However, the effect on Km is stronger than on Vmax, as shown by the reduced Vmax/Km ratio at 1 and 10 μM **2** relative to the control (Fig. S18D). These results indicate that the mechanism of action of **2** can be described by Figure S19, assuming α > 1. The maximum Km value (αKm) can be determined calculating the Km at saturating **2** concentrations from Fig. S19B, while the value of parameter a can be obtained from α = αKm/Km, yielding a value of 2.2. Similarly, the Vmax at saturating **2** concentrations (βVmax) was obtained from Fig. S19C, and the β value calculated from βVmax / Vmax ratio resulted to be of 2.0.

2,2-Diphenyl-1-picrylhydrazyl (DPPH) assay

The assay was performed according to a literature reported procedure. To 1.98 mL of a 200 μM DPPH solution in ethanol, 20 μL of a 20 mM ethanolic solution of compounds **1-3** and **8**, or dibutylhydroxytoluene (BHT) were added and rapidly mixed. The progress of the reaction was monitored by spectrophotometric analysis measuring the absorbance at 515 nm every 30 s for **10 min**. The percentage of DPPH inhibition was determined as follows:

$$Inhibition\ (\%) = \frac{A_0 - A_S}{A_0} \cdot 100$$

Where A_0: Absorbance of the control sample (DPPH in ethanol 100 %); A_S: Absorbance of the sample (solution of experimental sample in ethanol and DPPH in ethanol).

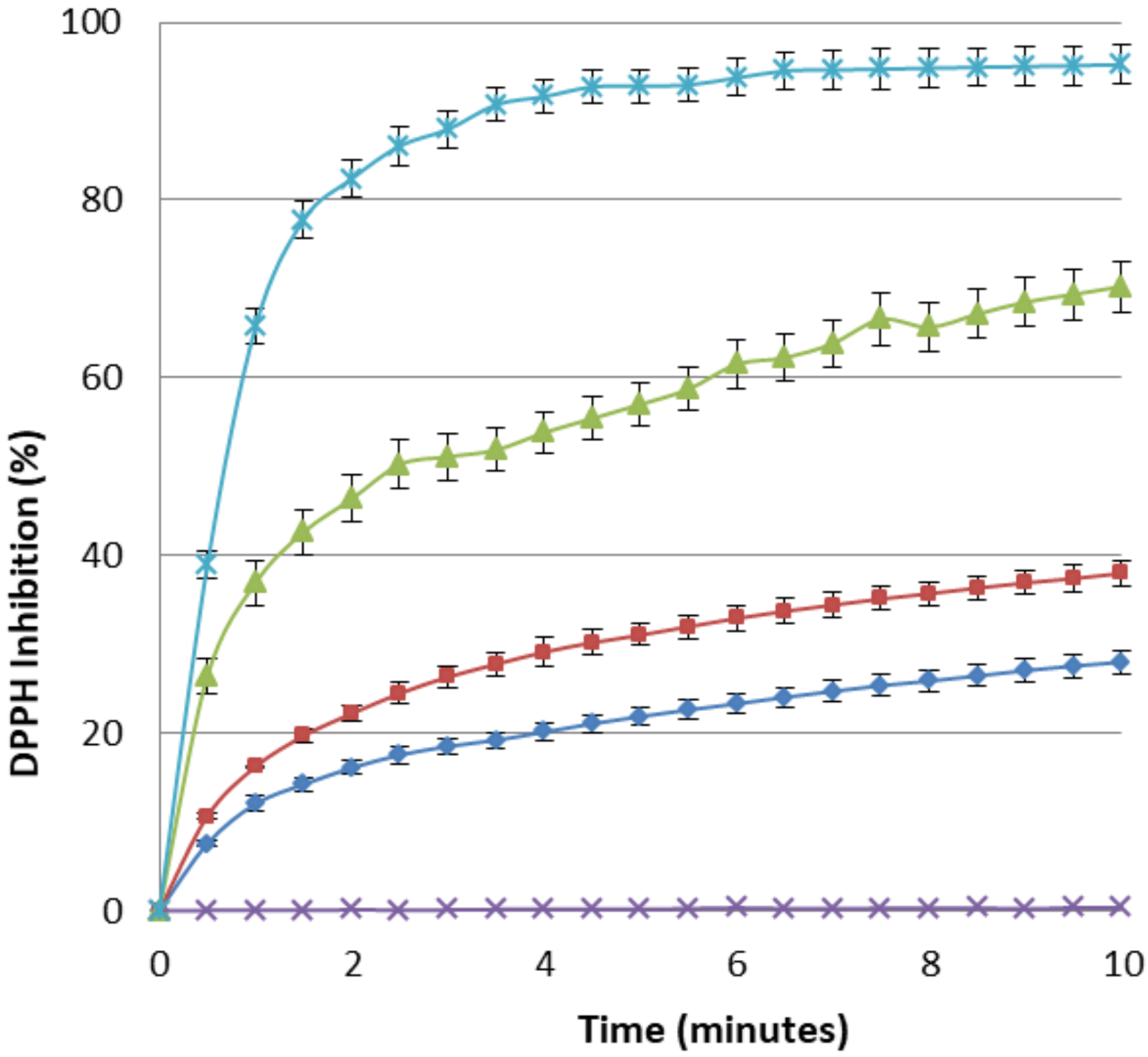

Figure S20: DPPH inhibition percentage in the presence of an equimolar amount of compounds **1** (green line), **2** (blue line), **3** (red line), **8** (purple line) and **BHT** (light blue). The mean ± SD values for three separate experiments are reported. Assay conducted on a 200 μM ethanolic solution of DPPH.

Chaperoning activity assays on Gaucher-derived fibroblasts

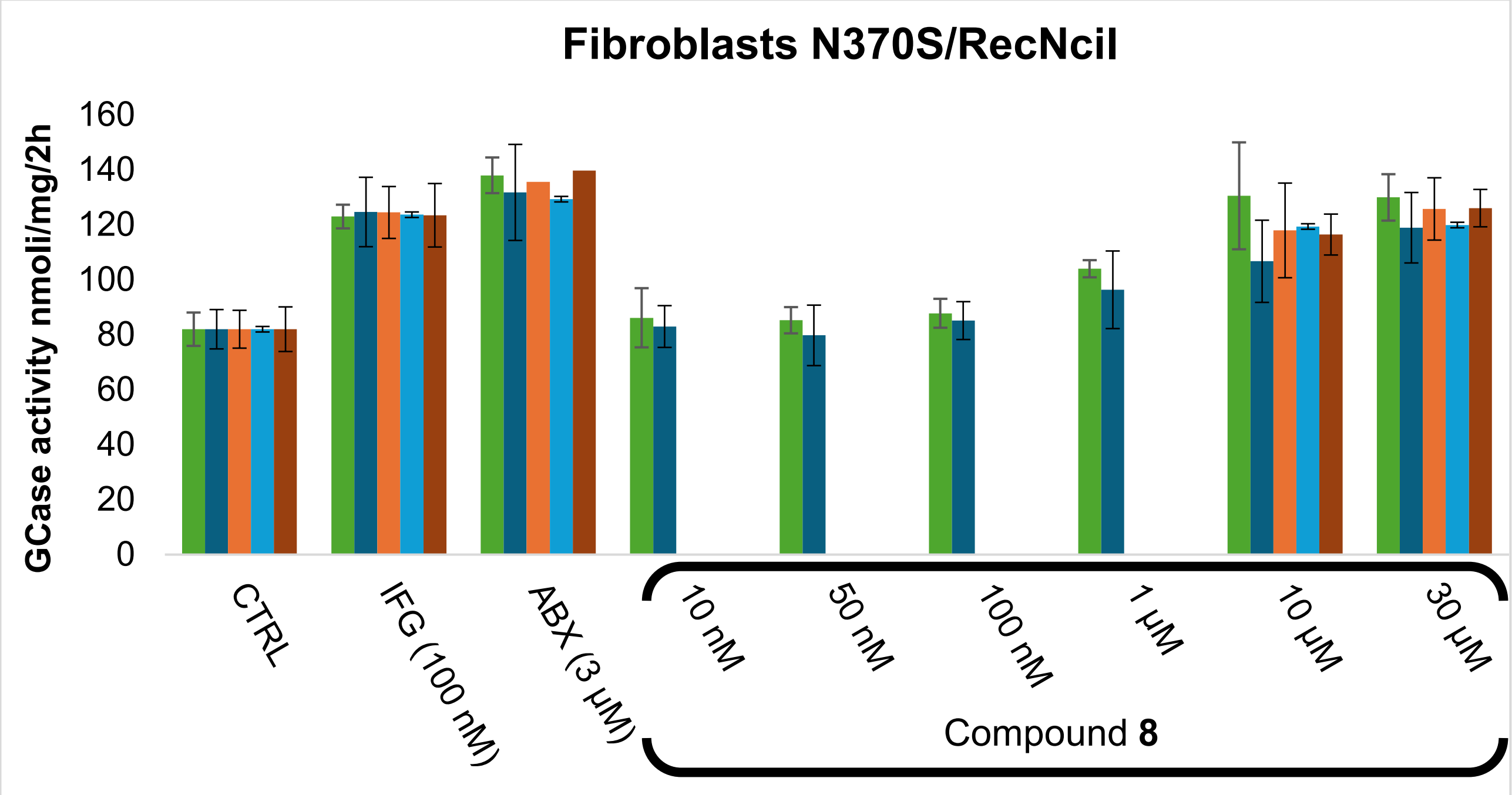

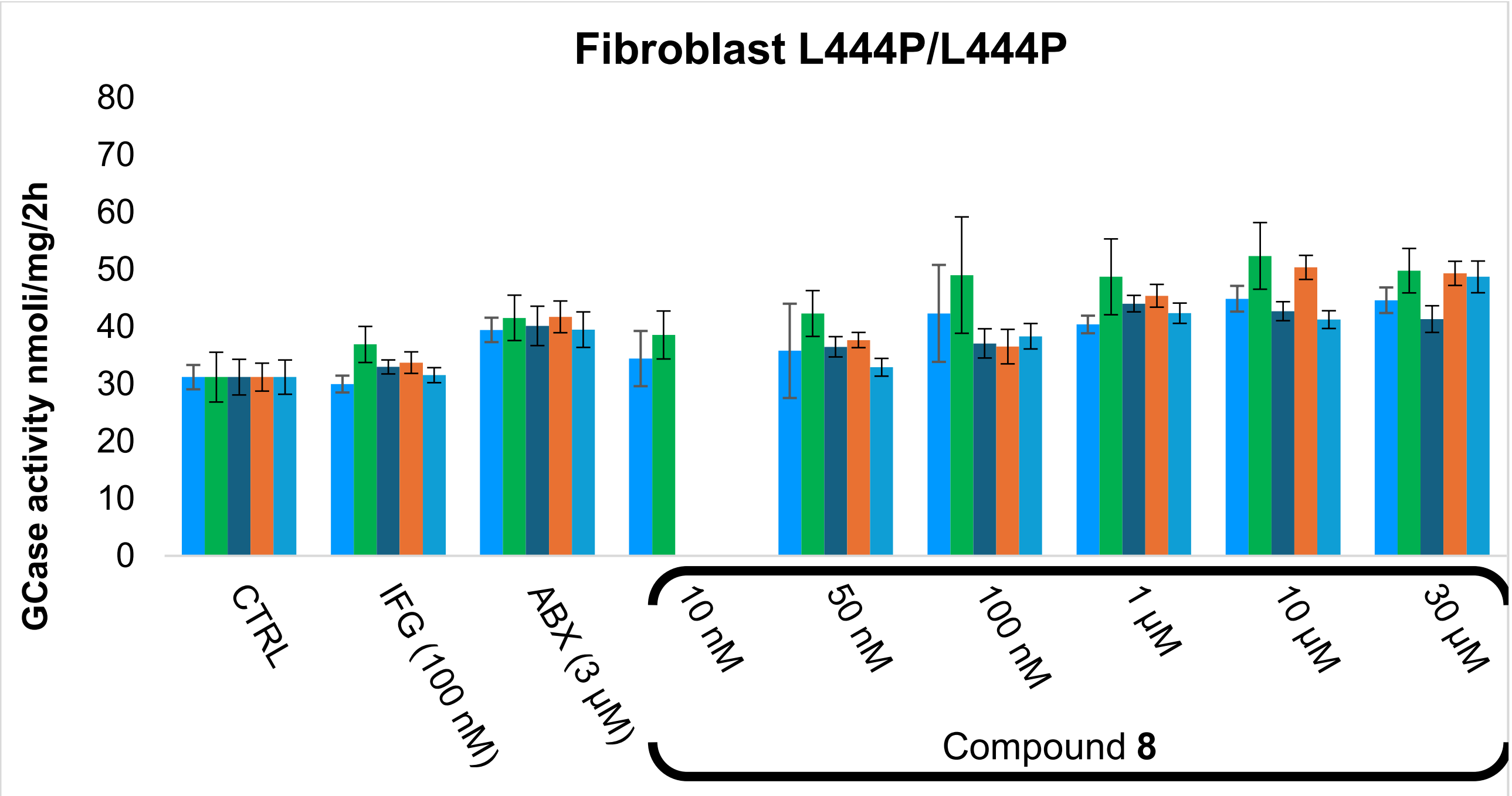

Figure S21: Fibroblasts derived from GD patients bearing A) N370S/RecNcil and B) L444P/L444P mutations were incubated without (control, CTRL) or with 6 different concentrations (10 nM - 30 μM) of compound **8** using isofagomine (IFG; 100 nM) and ambroxol (ABX; 3 μM) as control. After 4 days, the GCase activity was determined in intact cell as describe. The initial screening was performed in two independent biological experiments (n = 2). Selected concentrations showing the most relevant effects were subsequently evaluated in three additional independent experiments, resulting in a total of five independent biological experiments (n = 5) for these conditions. Untreated controls and compound-treated samples were analyzed in eight and four technical replicates, respectively, which were averaged within each independent biological experiment. Different colors represent independent experiments.

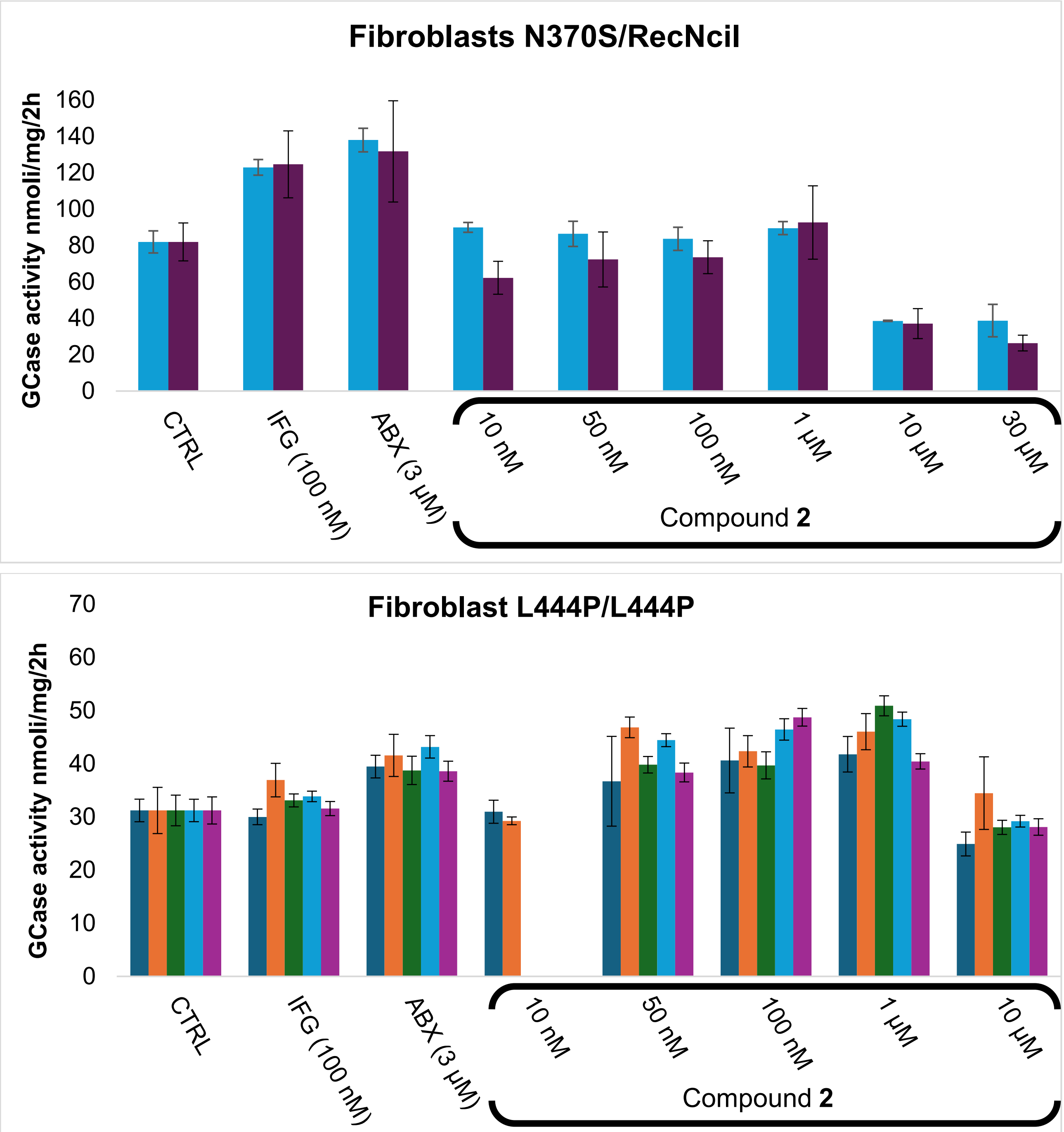

Figure S22: Fibroblasts derived from GD patients bearing A) N370S/RecNcil and B) L444P/L444P mutations were incubated without (control, CTRL) or with 6 different concentrations (10 nM -30 μM) of compound **2** using isofagomine (IFG; 100 nM) and ambroxol (ABX; 3 μM) as control. After 4 days, the flasks containing L444P/L444P mutation fibroblasts incubated with 30 μM concentration of **2** showed low cell viability. For the other concentrations, the GCase activity was determined in intact cell as describe. The initial screening was performed in two independent biological experiments (n = 2). Selected concentrations showing the most relevant effects were subsequently evaluated in three additional independent experiments, resulting in a total of five independent biological experiments (n = 5) for these conditions. Untreated controls and compound-treated samples were analyzed in eight and four technical replicates, respectively, which were averaged within each independent biological experiment. Different colors represent independent experiments.

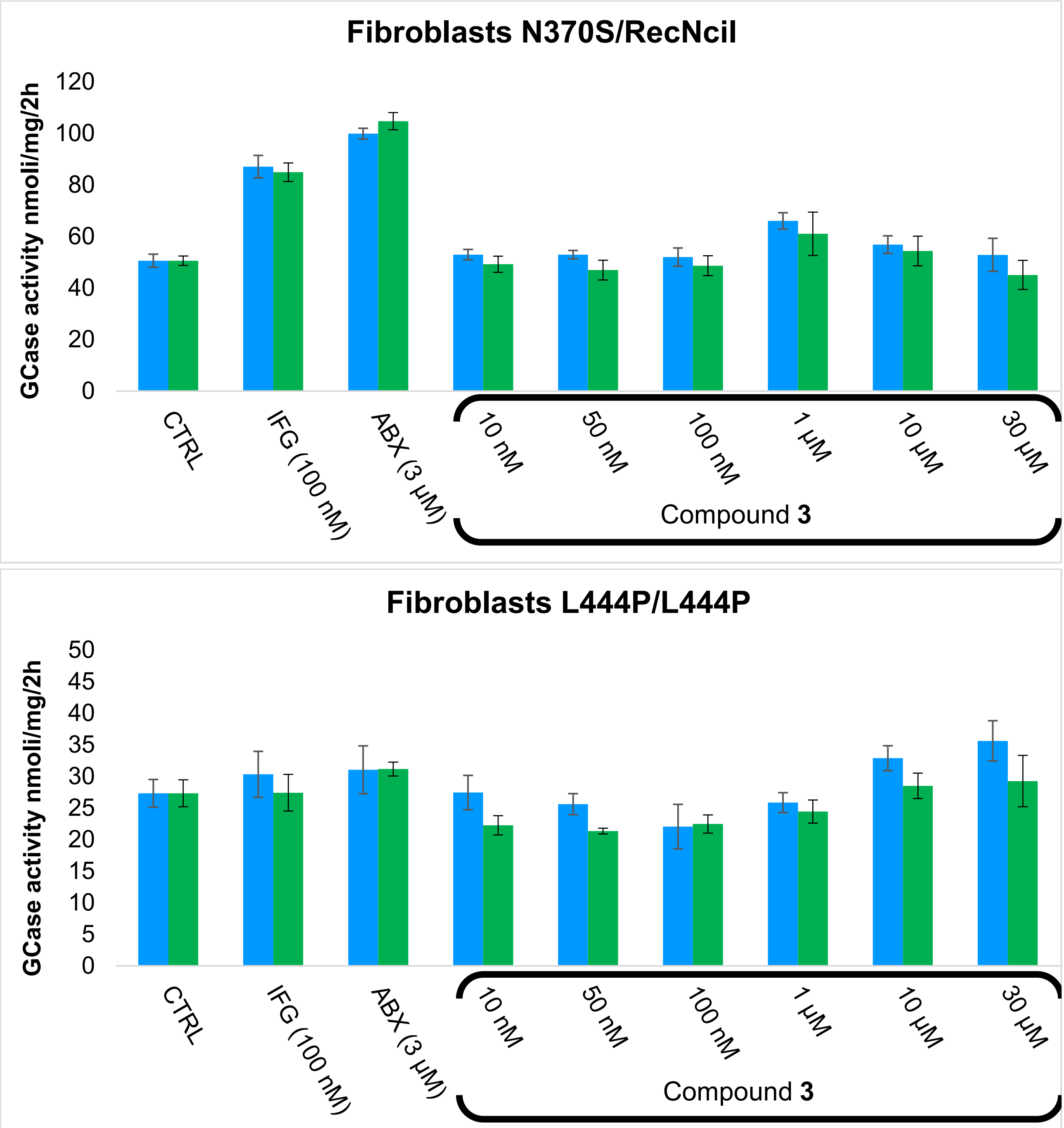

Figure S23: Fibroblasts derived from GD patients bearing A) N370S/RecNcil and B) L444P/L444P mutations were incubated without (control, CTRL) or with 6 different concentrations (50 nM -20 μM) of compound **3** using isofagomine (IFG; 100 nM) and ambroxol (ABX; 3 μM) as control. After 4 days, the GCase activity was determined in intact cell as describe. The initial screening was performed in two independent biological experiments (n = 2). Selected concentrations showing the most relevant effects were subsequently evaluated in three additional independent experiments, resulting in a total of five independent biological experiments (n = 5) for these conditions. Untreated controls and compound-treated samples were analyzed in eight and four technical replicates, respectively, which were averaged within each independent biological experiment. Different colors represent independent experiments.

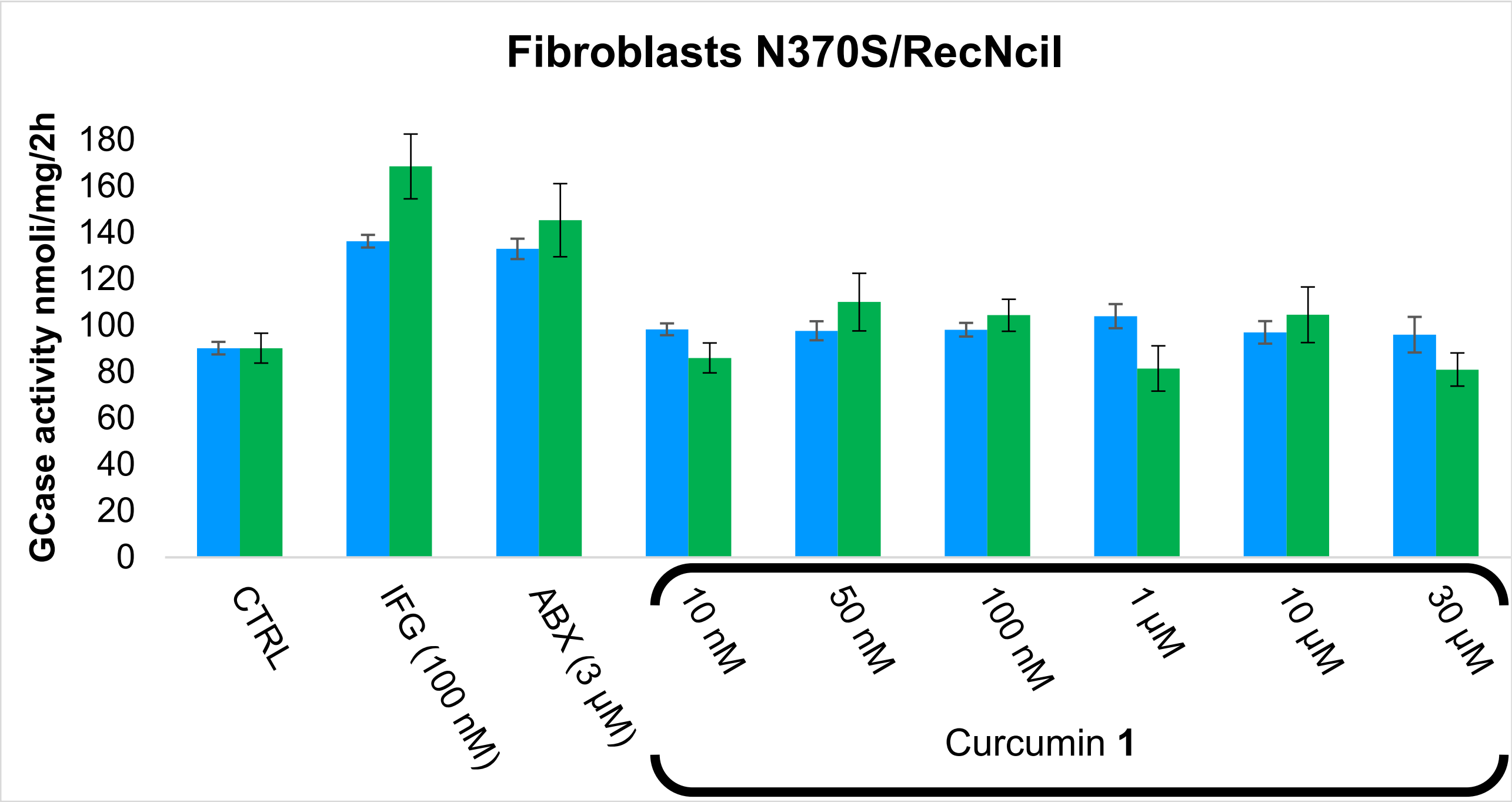

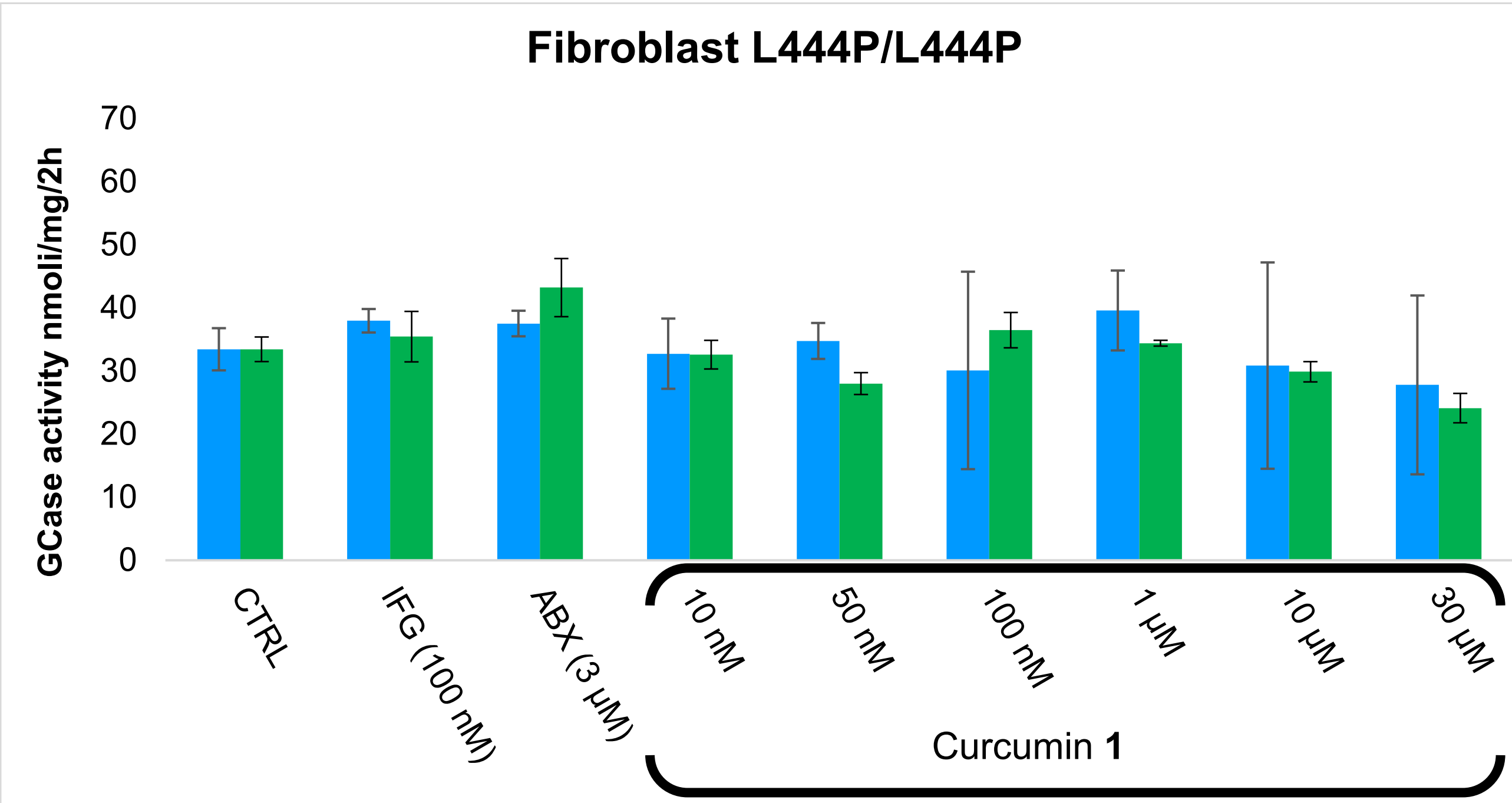

Figure S24: Fibroblasts derived from GD patients bearing A) N370S/RecNcil and B) L444P/L444P mutations were incubated without (control, CTRL) or with 6 different concentrations (10 nM -30 µM) of Curcumin **1** using isofagomine (IFG; 100 nM) and ambroxol (ABX; 3 µM) as control. After 4 days, the GCase activity was determined in intact cell as describe. The initial screening was performed in two independent biological experiments (n = 2). Selected concentrations showing the most relevant effects were subsequently evaluated in three additional independent experiments, resulting in a total of five independent biological experiments (n = 5) for these conditions. Untreated controls and compound-treated samples were analyzed in eight and four technical replicates, respectively, which were averaged within each independent biological experiment. Different colors represent independent experiments.

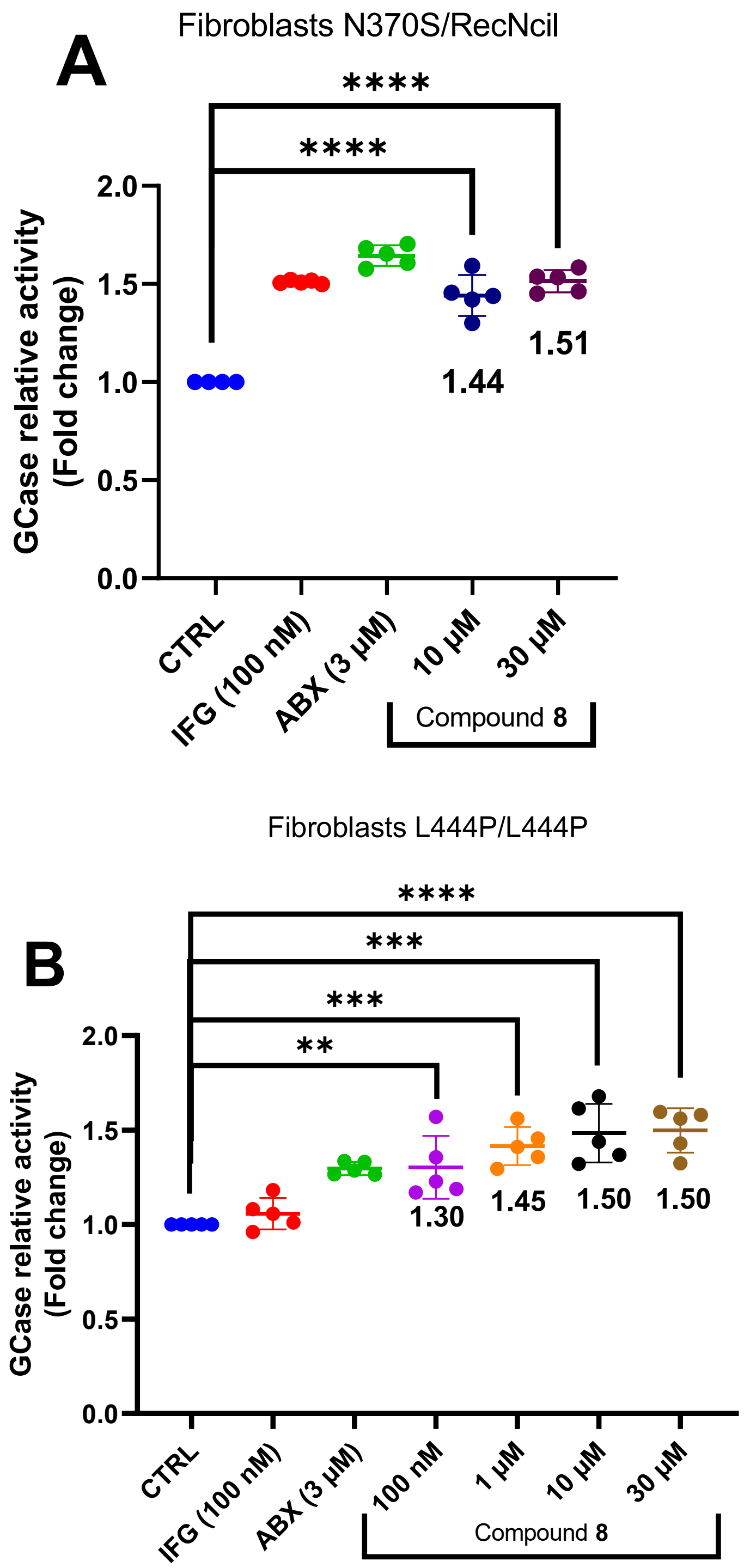

Figure S25: GCase relative activity is reported as the ratio between the GCase activity measured in human fibroblasts derived from GD patients with N370S/RecNcil (A) and L444P/L444P (B) mutations after 4 days of incubation with compound **8** and the GCase activity of the corresponding control without compound, using IFG (100 nM) and ABX (3 μM) as control. Data are presented as mean ± SD from five independent biological experiments (n = 5). Within each independent experiment, untreated controls and compound-treated samples were analyzed in eight and four technical replicates, respectively, which were averaged to obtain a single value per biological replicate. Statistical significance was determined using Student's t-test: * $p \leq 0.05$, ** $p \leq 0.01$, *** $p \leq 0.001$ and **** $p \leq 0.0001$.

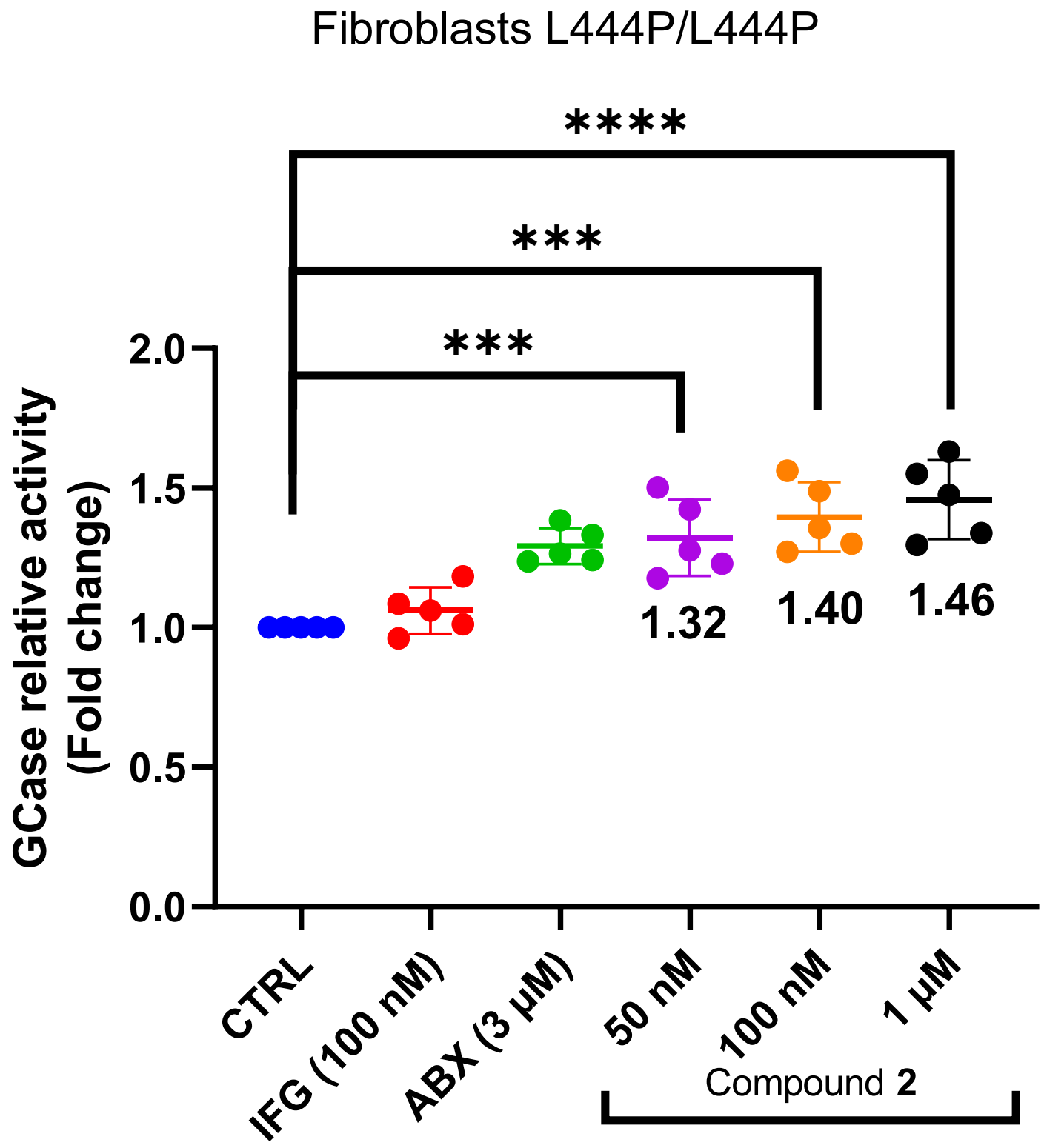

Figure S26: GCase relative activity is reported as the ratio between the GCase activity measured in human fibroblasts derived from GD patients with L444P/L444P mutation after 4 days of incubation with compound **2** and the GCase activity of the corresponding control without compound, using IFG (100 nM) and ABX (3 µM) as control. Data are presented as mean ± SD from five independent biological experiments (n = 5). Within each independent experiment, untreated controls and compound-treated samples were analyzed in eight and four technical replicates, respectively, which were averaged to obtain a single value per biological replicate. Statistical significance was determined using Student's t-test: * $p \leq 0.05$, ** $p \leq 0.01$, *** $p \leq 0.001$ and **** $p \leq 0.0001$.

Flow cytometric analysis of ROS levels in cells treated with compound 2

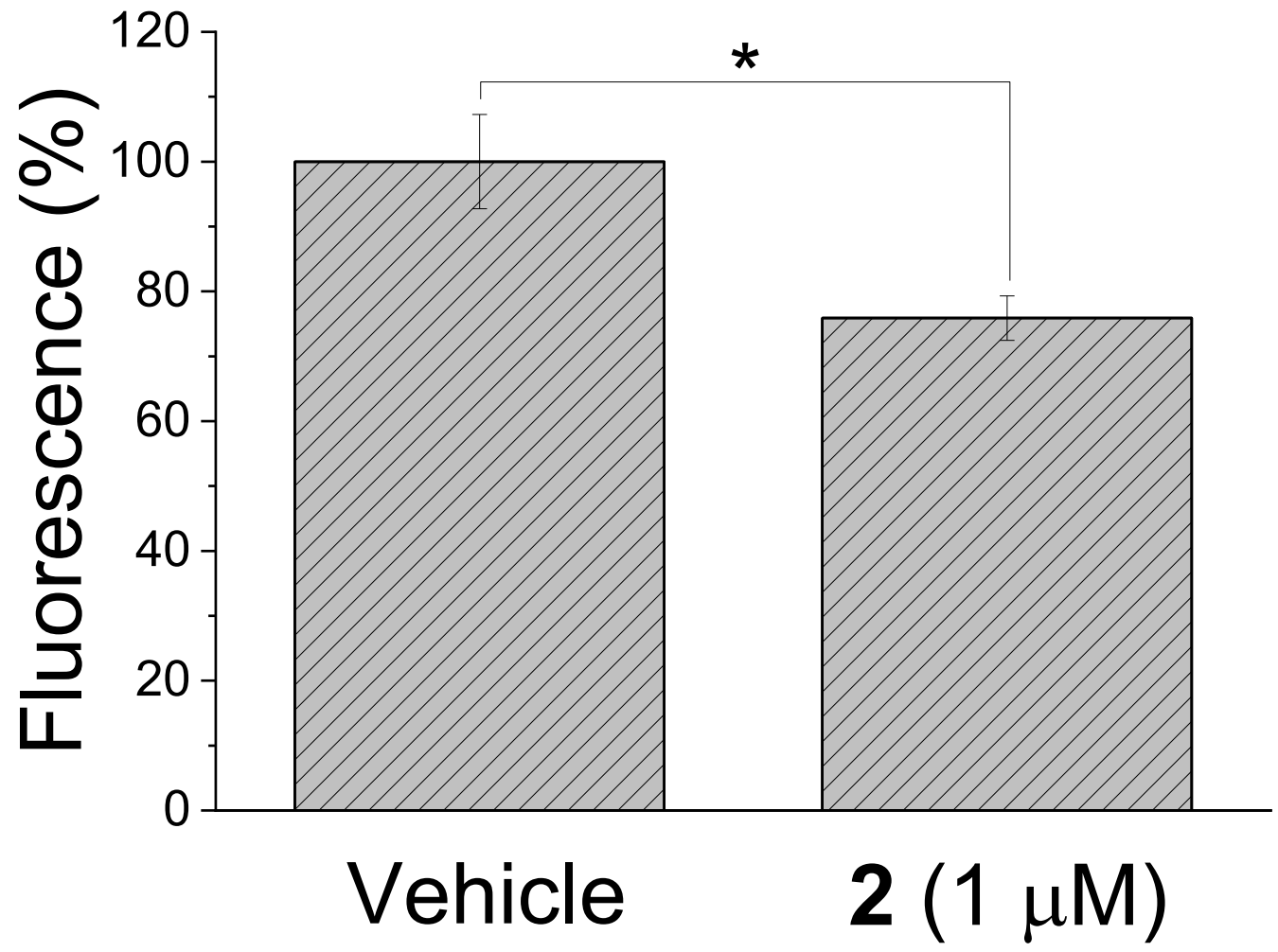

Figure S27: Effect of **2** on basal ROS level on fibroblasts derived from GD patients bearing L444P mutation.

Pharmacological Chaperoning Activity in Wild-Type Fibroblasts

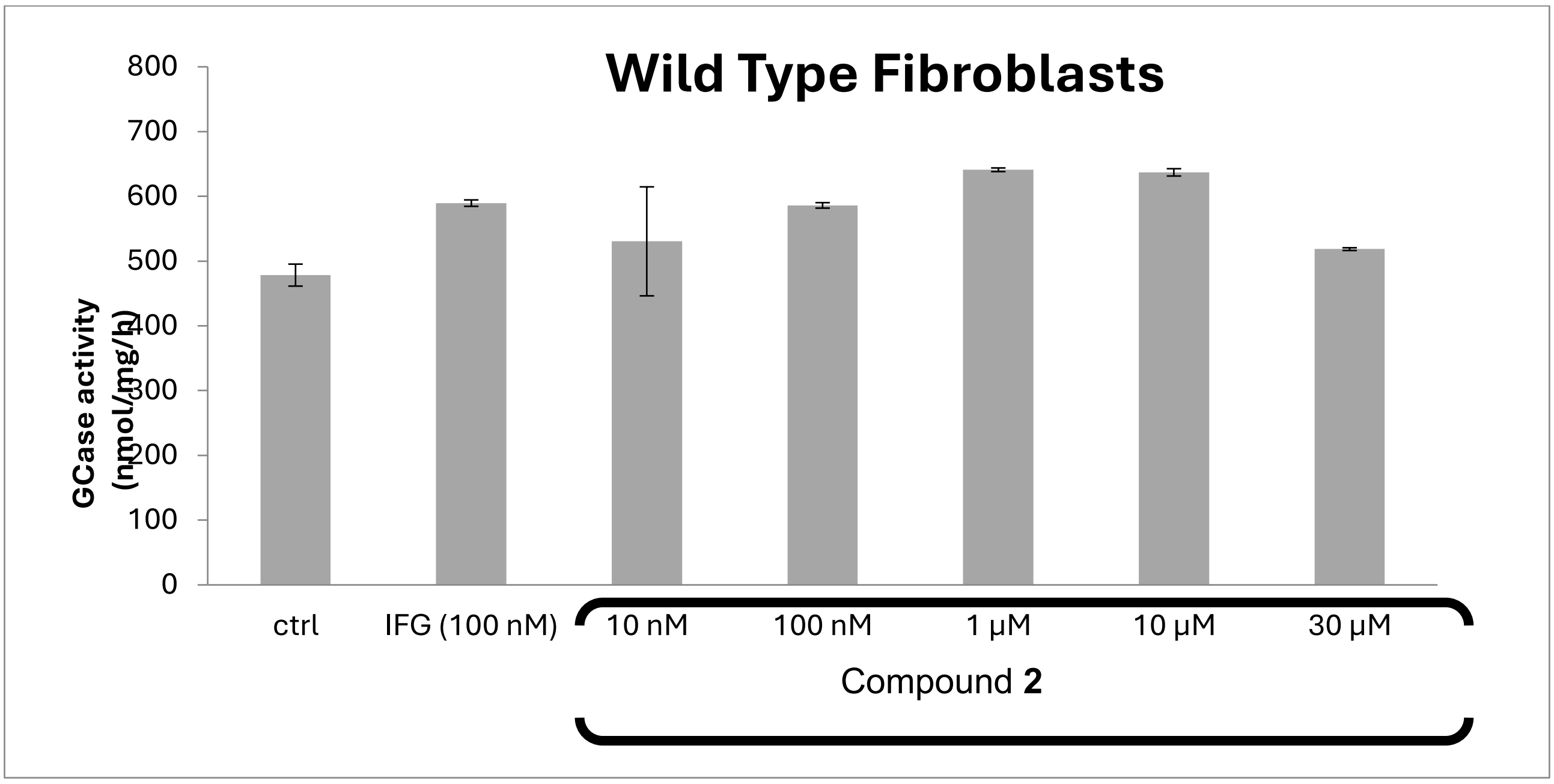

Figure S28: WT Fibroblasts were incubated without (control, ctrl) or with 6 different concentrations (10 nM - 50 µM) of compound **2,** using IFG (100 nM) as control. After 4 days, the flasks containing cells incubated with 50 µM concentration showed low cell viability that hampered to proceed with the assay. For the other concentrations, the GCase activity was determined (as above described) in lysates from treated fibroblasts. Data are reported as mean ± SD from two technical replicates (n = 2).

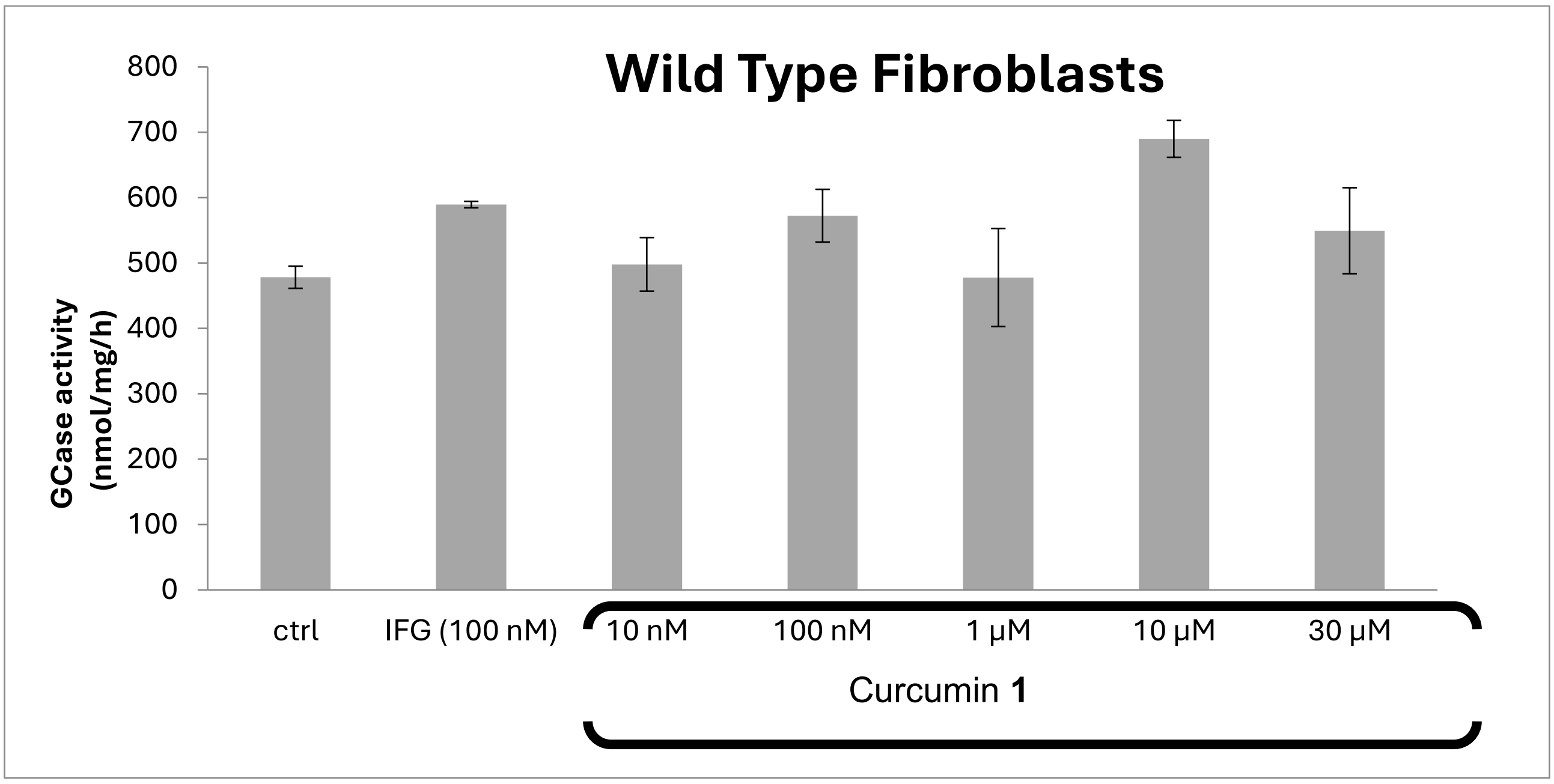

Figure S29: WT Fibroblasts were incubated without (control, ctrl) or with 6 different concentrations (10 nM - 50 µM) of curcumin **1,** using IFG (100 nM) as control. After 4 days, the flasks containing cells incubated with 50 µM concentration showed low cell viability that hampered to proceed with the assay. For the other concentrations, the GCase activity was determined (as above described) in lysates from treated fibroblasts. Data are reported as mean ± SD from two technical replicates (n = 2).
